# Fitness Landscape Transformation through a Single Amino Acid Change in the Rho Terminator

**DOI:** 10.1371/journal.pgen.1002744

**Published:** 2012-05-31

**Authors:** Lydia Freddolino, Hani Goodarzi, Saeed Tavazoie

**Affiliations:** 1 Joint Centers for Systems Biology, Columbia University, New York, New York, United States of America; 2 Department of Biochemistry and Molecular Biology, Columbia University, New York, New York, United States of America; University of Michigan, United States of America

## Abstract

Regulatory networks allow organisms to match adaptive behavior to the complex and dynamic contingencies of their native habitats. Upon a sudden transition to a novel environment, the mismatch between the native behavior and the new niche provides selective pressure for adaptive evolution through mutations in elements that control gene expression. In the case of core components of cellular regulation and metabolism, with broad control over diverse biological processes, such mutations may have substantial pleiotropic consequences. Through extensive phenotypic analyses, we have characterized the systems-level consequences of one such mutation (rho*) in the global transcriptional terminator Rho of *Escherichia coli*. We find that a single amino acid change in Rho results in a massive change in the fitness landscape of the cell, with widely discrepant fitness consequences of identical single locus perturbations in rho* versus *rho*^WT^ backgrounds. Our observations reveal the extent to which a single regulatory mutation can transform the entire fitness landscape of the cell, causing a massive change in the interpretation of individual mutations and altering the evolutionary trajectories which may be accessible to a bacterial population.

## Author Summary

Bacteria rely on complex genetic regulatory networks to respond to hazards or opportunities that they encounter. These networks consist of a series of sensory modules, coupled with various response elements that must be appropriately activated to deal with a given set of environmental conditions; all of these condition-specific elements interact with the cell's core machinery for gene expression. When they encounter a novel environment, populations of bacteria rapidly evolve to adapt to that environment; alterations in gene expression play a major role in this process and, in particular, mutations to the cell's central gene expression machinery are surprisingly common in laboratory evolution experiments. Focusing on one such mutation that had previously been shown to enhance the host cell's ethanol tolerance, we show that the same alteration can in fact aid cellular survival under a wide variety of conditions. In addition, the interactions of this regulatory mutation with other genes throughout the genome cause these mutations to fundamentally reshape the effects of any other genomic changes that occur, and thus alter the overall evolutionary course taken by a population.

## Introduction

Rho-dependent termination is a crucial component of transcriptional regulation in bacteria, and is estimated to terminate approximately half of the transcripts present in *E. coli* [1], [2]. Recent studies have shown that this type of transcription termination is particularly prevalent in prophage and other horizontally acquired DNA, thus insulating the cell from the deleterious expression of such elements [3], [4]. Rho has also been shown to safeguard genomic integrity by reducing co-directional collisions between transcriptional and replication machinery [5], [6]. The rho* allele was initially identified in a set of short-term laboratory evolution experiments as a major modifier of ethanol tolerance in *E. coli* MG1655 [7]. This allele contains a missense mutation (F62L) in the RNA binding domain of Rho, which has been previously shown to cause a 20% higher read-through of the termination site tR1 [8], and raise the dissociation constant for (rC)_10_ by a factor of four [8]. The ethanol tolerance caused by rho* can be traced to overexpression of a few loci (namely the *prpBCDE* and *cadBA* operons [9]), which are also among the transcriptional units strongly affected by chemical inhibition of Rho-dependent termination [3]. Mutations to *rho* have also been observed in several other laboratory evolution experiments [10]–[13], although the nature of their contribution to fitness in those cases is unclear.

Given the pervasive effects on transcription throughout the genome caused by short term inhibition of Rho-dependent termination [3], [4], we sought to determine the full breadth of effects of rho*, both on cellular phenotype and on secondary mutations at other loci. We found widespread effects from both classes; rho* significantly alters cellular fitness in the presence of a variety of nutrient sources and antibiotics, and shows epistatic interactions with mutations at ∼5% of other loci in the genome. Our results illustrate that mutations to rho*, and presumably other central components of the transcriptional apparatus, facilitate the rapid generation of broad phenotypic diversity in bacteria, with significant consequences for the evolution of populations under stress.

## Results

### rho* causes diverse, bidirectional changes in transcript levels

Based on the biological function of Rho, one naturally expects that rho* cells will show increased transcription immediately downstream of Rho-dependent termination sites. Indeed, measurements of transcript abundances [3] and RNA polymerase occupancy [4] have recently shown that after short-term inhibition of Rho-dependent termination using the compound bicyclomycin (BCM), hundreds of transcriptional readthrough events are apparent throughout the *E. coli* genome, with significant over-representation of recently and horizontally acquired genomic regions. In order to assess the effects of rho* on transcriptional output during balanced growth, we performed transcriptional profiling comparing WT and rho* cells using tiling microarrays (raw data available at the Gene Expression Omnibus; Accession GSE32022). We then identified genomic regions showing significant differences in transcript levels between the two genetic backgrounds (Bonferroni-corrected p<0.01 and greater than twofold change in representation; see Text S1, Section 1.6). We found a total of 2535 probes (out of 92794 positions) showing significant differences, located in 1281 genes and 433 intergenic regions; a few example loci are shown in Figure S1. We identified the most significantly perturbed genes in rho* by flagging all cases for which the median WT:rho* expression ratio for all sense-stranded probes in a given gene indicated a greater than 1.5-fold change in expression level; using this threshold, 155 genes were overexpressed and 44 underexpressed in rho*. The presence of such a substantial underexpressed fraction again illustrates the presence of indirect effects of rho* on the genetic regulatory network, whereas the overexpressed fraction likely represents a combination of genes overexpressed directly due to transcriptional readthrough and those altered due to regulatory perturbations. Consistent with this interpretation, probes which are significantly overexpressed in rho* cells relative to WT show 1.3-fold enriched overlap with a set of prophages, insertion sequences, and K-12 specific elements (the MDS42 deletion sites [14]; p = 0.011 by random permutation of site locations). Probes overexpressed in WT cells, in contrast, show no significant correlation with MDS42 deletion sites (1.2-fold depletion; p = 0.198).

It is also notable that of the probes identified as significantly overexpressed in rho* cells relative to *rho*^WT^, 82% of those overlapping genes were on the antisense strand (*cf.* 55% for those underexpressed in rho*; see Figure S2A). A recent RNA-seq study showed the presence of pervasive antisense transcription throughout the *E. coli* genome [15], which the authors presume to be limited in extent primarily by Rho-dependent termination [15]. In addition, Peters and coworkers identified 24 novel antisense transcripts appearing in BCM-treated cells [4], more directly illustrating a role for Rho-dependent termination in at least some cases. In order to assess the effects of rho* on previously identified antisense transcripts, we compared the log-ratios of transcript levels in rho* vs *rho*^WT^ cells along a series of windows centered at 50 bp increments downstream of the 1,005 antisense transcription start sites identified by Dornenburg *et al.* [15]. As seen in Figure S2B, a significant increase in transcription is apparent in rho* cells along the first several hundred bp of these antisense transcripts, illustrating a major mechanism through which rho* likely alters cellular physiology. Furthermore, this analysis does not capture the effects on antisense transcripts which are at undetectable levels in *rho*^WT^ cells (and thus would have been missed from the Dornenburg study).

**Figure 1:**
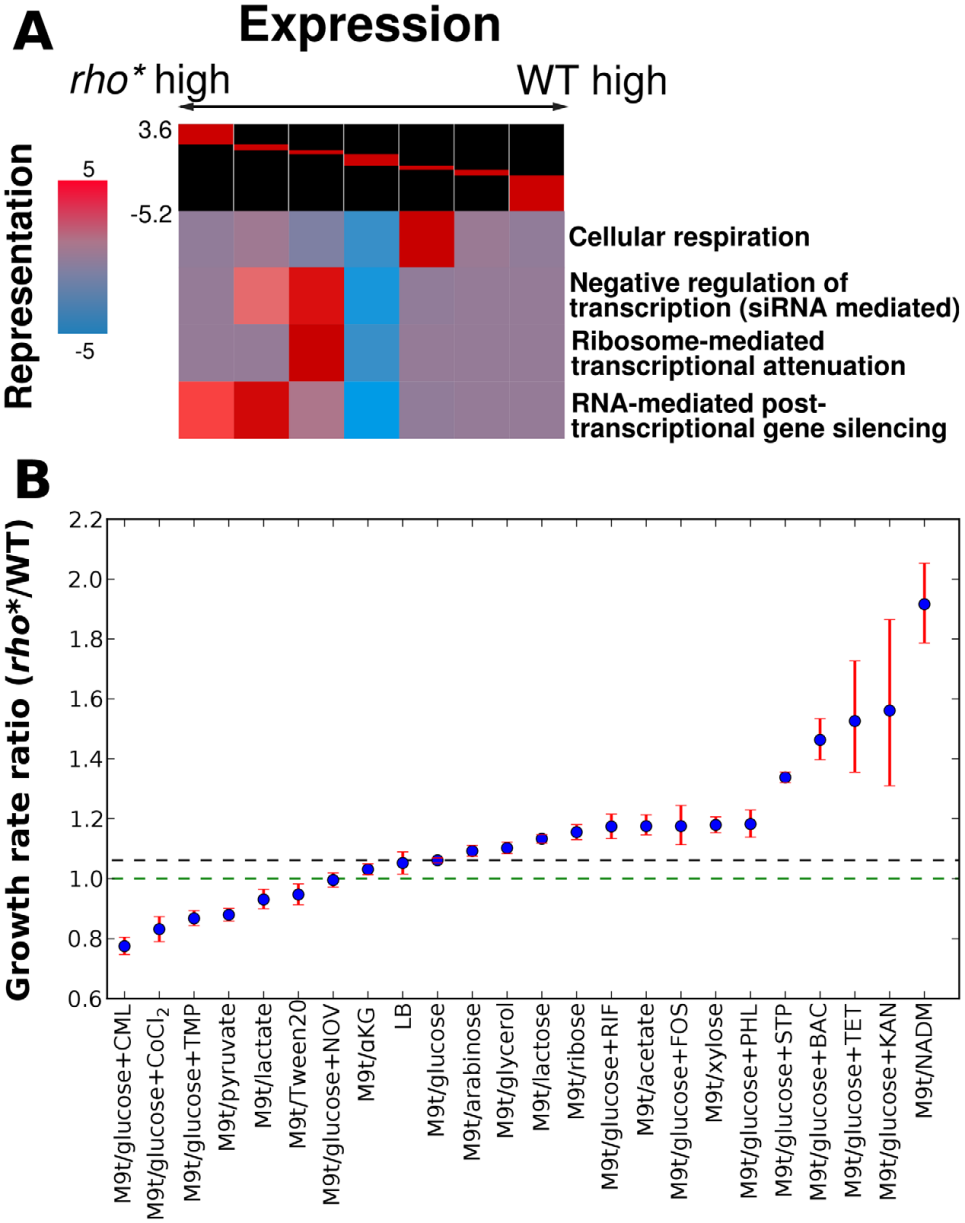
Comparison of transcriptional and phenotypic differences between WT and rho* cells. (A) Key pathways showing significant patterns of over- or under-expression in rho* cells, assessed using iPAGE [16]. The log ratios of rho* to WT RNA at each probe were discretized as shown at the head of each chart (breaks correspond to 3, 2, and 1.5-fold differences in either direction). For each pathway, coloring at each cluster corresponds to the degree of over- or under-representation of that cluster in the relative expression level of genes in that pathway (with magnitude equal to the negative log_10_ p-value for significance of the representation; positive and negative scores represent over- and under-representation, respectively). (B) Ratios of rho* to WT growth rates under a variety of conditions showing significant fitness differences; dashed lines indicate the ratio under the reference condition (black) or for equal growth rates (green). Error bars show a 95% confidence interval based on 10,000 samples from the posterior parameter distribution (see Text S1, Section 1.3). Abbreviations are shown in Table S10. See Table 1 for concentrations of antibiotics used. Ratios of rho* to WT growth rates differ significantly from that in the reference condition M9t/glucose (non-overlapping 95% confidence intervals) for all cases except LB, which is included simply as a second useful reference.

In order to obtain a pathway-level view of the changes in gene expression caused by rho*, we applied iPAGE [16] to identify gene ontology (GO) pathways which share significant mutual information with the log-ratio of rho* vs. WT RNA from microarray experiments (see Text S1, Section 1.7 for details). In all, 19 non-redundant GO terms show significant mutual information with the expression profile for sense-strand RNA and 10 non-redundant GO terms for the antisense profile (out of 1340 present in the annotation set [17], using a threshold of p<0.0001). The changes in expression patterns for a few example pathways of particular interest are shown in Figure 1A, and the full set of significantly perturbed GO terms is shown in Figure S3. These changes in expression affect a variety of cellular pathways including diverse aspects of metabolism and regulation; for example, genes involved in transcriptional attenuation and post-transcriptional regulation were over-expressed in the rho* background, which may represent a regulatory coping strategy for minimizing the deleterious effects of transcriptional read-through. For the most part, however, the fitness consequences (if any) of these broad-reaching expression modifications were not readily identifiable.

### rho* shows direct fitness effects under a wide variety of conditions

In order to measure the extent to which the altered gene expression state of rho* MG1655 cells affects their fitness in different environments, we compared the growth of these cells to that of wild type cells in the presence of a variety of nutrient conditions and antibiotics (see Text S1, Section 1.2 for details). We identified 22 conditions (shown in Figure 1B and Table 1) in which the relative fitness of WT and rho* differed significantly from that in our reference condition (glucose minimal media), with 8 conditions favoring WT and 14 favoring rho* cells (we use steady-state growth rate as a proxy for fitness unless otherwise noted; see Section 2 of Text S1, Table S1, and Figure S4 for a discussion of other relevant quantities). The number and nature of these discovered environments show that the regulatory perturbations caused by the rho* mutation functionally modify a variety of pathways in the cell. In some cases the fitness differences between WT and rho* cells can be directly explained by modified gene expression. For example, the pathway-level analysis in Figure 1A shows that pathways involved in oxidative metabolism are under-expressed in the rho* background, which may explain their increased aminoglycoside resistance [18]. Most conditions showing fitness differences, however, defy such simple explanations.

**Table 1:** Conditions showing significant differences in growth rate between WT and rho* cells (relative to growth in M9t/glucose).

Media	WT value (95% CI)	rho* value (95% CI)
M9t/acetate	0.269 (0.258–0.279)	0.316 (0.304–0.329)
M9t/**α**KG	0.509 (0.489–0.531)	0.521 (0.504–0.548)
M9t/arabinose	0.947 (0.913–0.981)	1.034 (0.996–1.074)
M9t/glucose	1.017 (0.996–1.038)	1.079 (1.057–1.101)
M9t/glucose+CML1.875	0.379 (0.287–0.499)	0.294 (0.223–0.386)
M9t/glucose+STP 2.0	0.770 (0.7329–0.808)	1.030 (0.980–1.081)
M9t/glucose+NOV 150.0	0.867 (0.845–0.888)	0.862 (0.841–0.883)
M9t/glucose+TMP 0.5	0.287 (0.268–0.308)	0.249 (0.232–0.267)
M9t/glucose+CoCl_2_ (2.5 µM)†	0.781 (0.689–0.885)	0.648 (0.571–0.735)
M9t/glucose+FOS 2.5†	0.434 (0.388–0.486)	0.511 (0.456–0.572)
M9t/glucose+RIF 1.5	0.870 (0.823–0.920)	1.021 (0.966–1.081)
M9t/glucose+PHL 0.5	0.802 (0.777–0.829)	0.948 (0.919–0.979)
M9t/glucose+BAC 100†	0.742 (0.714–0.769)	1.085 (1.045–1.125)
M9t/glucose+TET 0.5†	0.215 (0.167–0.280)	0.321 (0.249–0.416)
M9t/glucose+KAN 2.0†	0.704 (0.599–0.829)	1.099 (0.934–1.295)
M9t/glycerol	0.577 (0.552–0.603)	0.636 (0.607–0.665)
M9t/lactate	0.571 (0.556–0.587)	0.532 (0.516–0.548)
M9t/lactose	0.967 (0.947–0.987)	1.095 (1.071–1.119)
LB	2.510 (2.345–2.688)	2.640 (2.469–2.828)
M9t/pyruvate	0.563 (0.555–0.572)	0.495 (0.486–0.504)
M9t/ribose	0.480 (0.462–0.498)	0.554 (0.533–0.575)
M9t/Tween20	0.253 (0.241–0.266)	0.240 (0.227–0.252)
M9t/xylose	0.744 (0.727–0.761)	0.877 (0.854–0.900)
M9t/NADM†	0.049 (0.045–0.054)	0.094 (0.086–0.104)

Growth rates of selected strain/condition combinations are defined as described in the Methods section of the main text. All growth rates are in doublings/hour and include 95% confidence intervals from sampling of the posterior model parameter distributions (see Text S1, Section 1.3). Significance of differences was assessed relative to growth in M9t/glucose as described in the caption for Figure 1. Growth in LB did not differ significantly from growth in M9t/glucose, but is included as a useful reference point. Numbers of technical replicates used for each growth rate are given in Table S11. Abbreviations are listed in Table S10.

† : Effective growth rate; see Text S1 for details. Concentrations of additives are given in µg/mL unless otherwise noted.

### rho* interacts with secondary mutations throughout the genome

**Figure 2:**
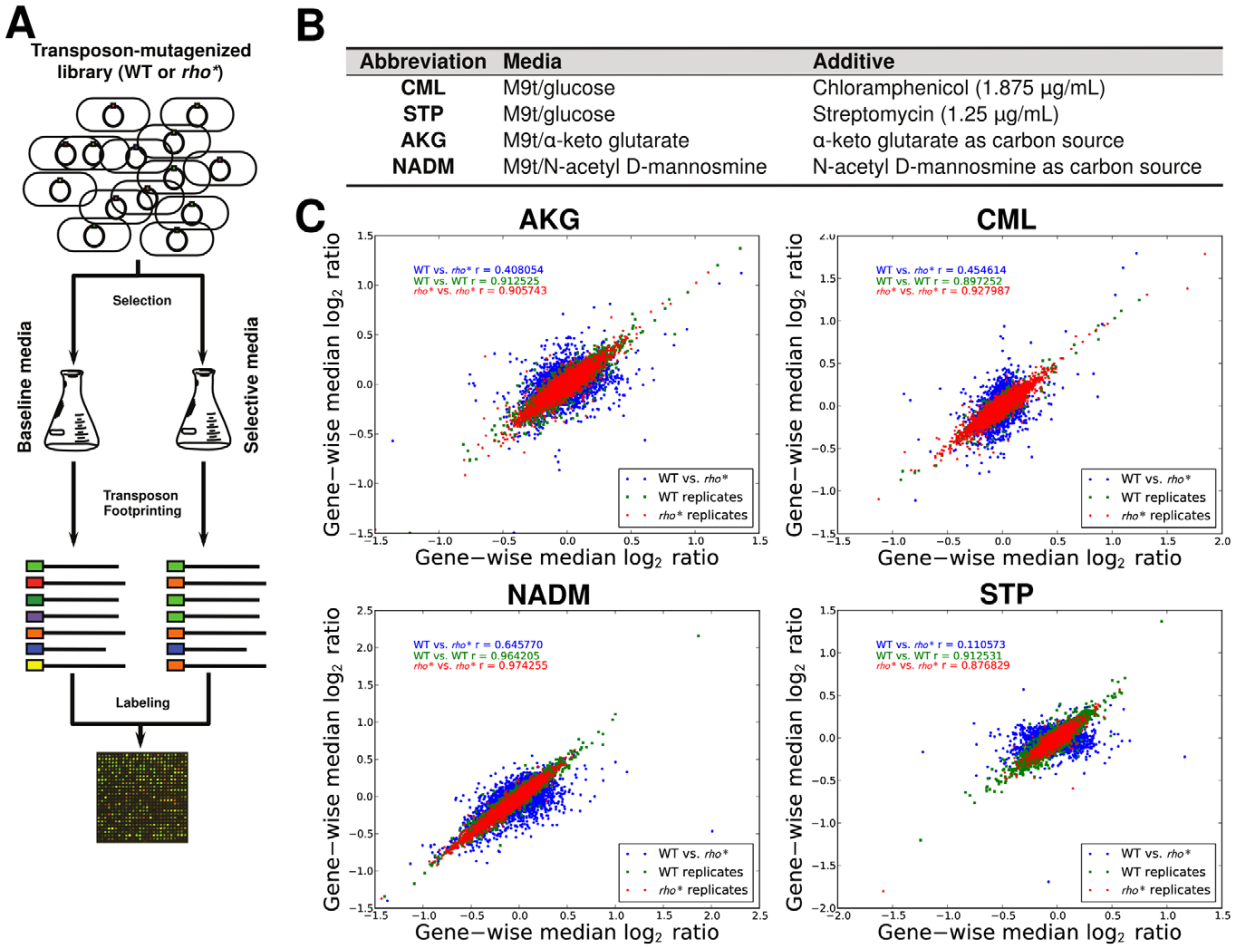
Mapping of fitness landscapes of WT and rho* cells using transposon-mutagenized libraries. (A) Procedure used to identify loci with significant fitness contributions. Transposon mutagenized libraries were constructed separately in the WT and rho* strains, and then grown in parallel under selective conditions and a reference condition. Genomic regions adjacent to transposons were then selectively amplified. The abundances of transposon insertions throughout the genome were compared between the condition of interest and the reference condition using a two-color microarray. (B) Selective conditions used; M9t/glucose was the reference condition in all cases. (C) Comparison of gene-wise median fitness scores (log_2_ ratio of reference∶selected microarray signals) between WT and rho* cells under each condition. For reference, a similar comparison between the two biological replicates of each strain/condition pairing is also shown. Pearson correlations (r) are given between WT and rho* signals or between replicate experiments for isogenic cells, as appropriate.

**Figure 3:**
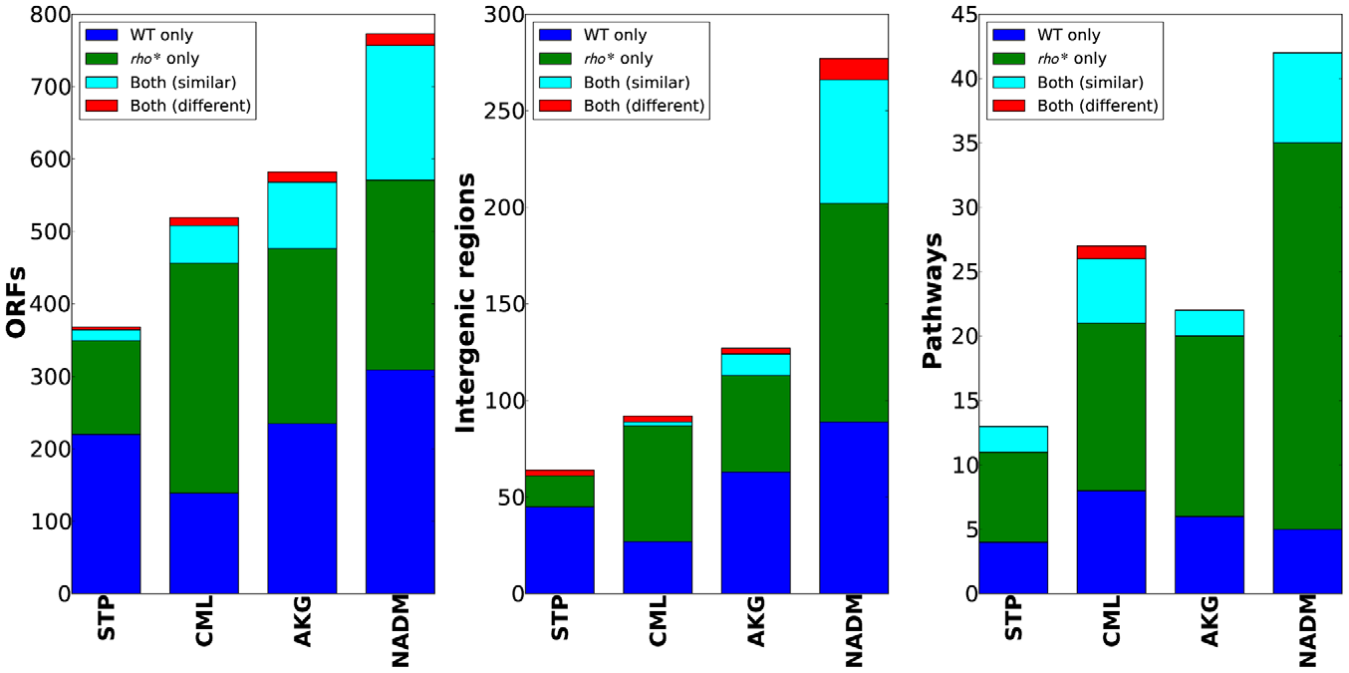
WT and rho* backgrounds show remarkably different fitness landscapes. Bar charts show, from left, the number of genes, intergenic regions, and pathways identified as having informative over- or under-representations of transposon insertions (see main text and Text S1, Section 1.5 for details). Categories which appeared as significant in both WT and rho* data are further subdivided based on whether the pattern of enrichment and depletion was correlated in the two backgrounds.

**Figure 4:**
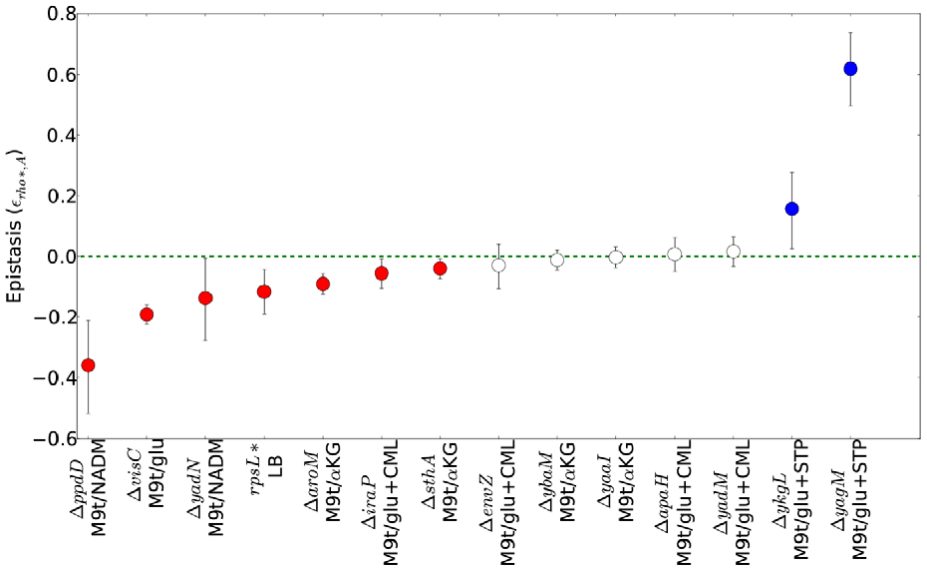
Epistatic interactions between rho* and secondary mutations. For each combination of a secondary mutation *A* and media condition (shown on the *_x_* axis) considered in our follow-up experiments, the multiplicative epistasis ε_rho*,A_ (see Eq. S7) is plotted, along with error bars showing a 95% confidence interval (see Text S1, Section 1.3). Significant interactions (those for which the confidence intervals do not overlap with zero) are colored according to their sign. Positive values of ε_rho*,A_ indicate positive epistasis between rho* and the secondary mutation in question (*i.e.*, the fitness of the double mutant is higher than that expected based on the effects of the two mutations in isolation). Complete data for all strains are given in Table S4. rpsL* strains in LB+ethanol are not shown due to the complete absence of wild type growth in this condition.

The varied, pleiotropic effects of rho* on fitness under different growth conditions suggested that the rho* mutation may also result in global changes in the fitness landscape, altering the effects of any additional mutations. To test for such changes, we used fitness profiling of transposon-mutagenized libraries [19] to create coarse-grained representations of the fitness landscape under four conditions (a schematic of the procedure is shown in Figure 2A–2B and detailed methods are provided in Text S1, Section 1.5; raw data are available from the Gene Expression Omnibus, Accession GSE32022). For a given condition, a modified fitness landscape implies that there are loci whose fitness consequences are different in *rho*^WT^ and rho* backgrounds. These loci, in turn, provide insight into the specific mechanisms through which rho* alters the cell's regulatory and physiological state (we provide more detailed analysis of several such cases, including follow-up experiments on knockout strains, in Text S1, Section 3; see also Figures S5 and S6 and Table S2). Similar patterns emerged in all four conditions tested: both the WT and rho* fitness profiles show hundreds of sites at which transposon insertions lead to significant changes in fitness, with the majority unique to one genetic background or the other (see Table S3). Comparisons of the distributions of selection scores between WT and rho* cells in each condition are shown in Figure 2C; the low correlations between scores of genes in the two genetic backgrounds under all four conditions indicate that the fitness consequences of secondary mutations are heavily dependent on the genotype at the *rho* locus, whereas correlations between replicates from the same genetic background under each condition are quite high. The overlaps of loci and pathways with significant fitness effects in the two backgrounds are shown in Figure 3; in all four cases, a common core of loci exists which strongly contributes to fitness in both the *rho*^WT^ and rho* backgrounds, but the majority (>70%) are unique to one background or the other. This indicates that the effects of these mutations are in fact strongly altered by the rho* allele. Consistently, in Figure 4 we show examples of several loci where significant epistasis between rho* and a secondary mutation was observed in defined strains during follow-up experiments (details of the epistasis experiments and calculations are given in Text S1, Section 4; see also Tables S4 and S5).

**Figure 5:**
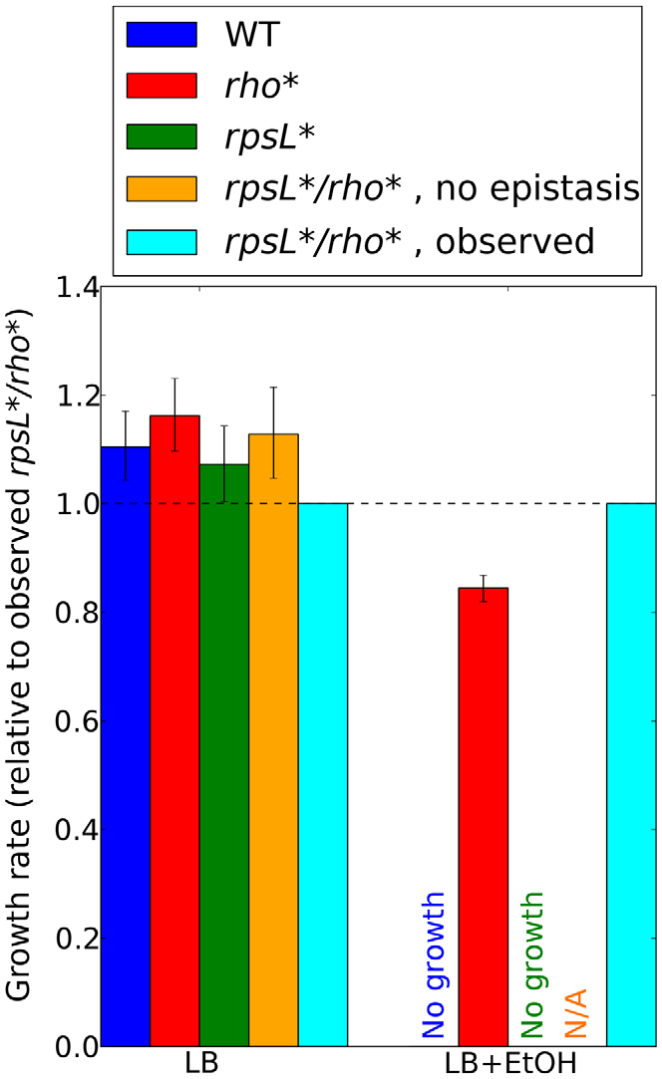
Contributions of rho*-rpsL* epistatic interactions to ethanol tolerance. Ratios of growth rates for each strain to those for the rho*/rpsL* double mutant are shown for all permutations of mutant and wild type alleles at these loci, in LB (left) or LB plus 5.5% ethanol (right). See Section 5 of Text S1 for details on growth rate determination in ethanol-containing media. Error bars indicate 95% confidence intervals. Predictions for the double mutant in the absence of epistasis are made using a multiplicative model (see Text S1, Section 4). Such predictions are not possible in LB+EtOH due to the lack of *rho*^WT^ and rpsL* growth.

The genetic basis of laboratory-evolved ethanol tolerance provides an example of the reshaping of the fitness landscape by rho*. In the course of the experiments reported here, we found that rho* alone is insufficient to confer the levels of ethanol tolerance observed in the evolved strain from [7]. Instead, using global linkage analysis, we found that an epistatic interaction between rho* and rpsL* (a nonsense mutation in the S12 ribosomal protein RpsL) provides a substantial portion of the increase in ethanol tolerance (see Section 5 of Text S1, Figures S7 and S8, and Table S6 for further details). Relative growth rates for all combinations of wild type and identified mutant alleles of rho* and rpsL* are shown in Figure 5. Whereas the rpsL*/rho* double mutant showed a maximum growth rate of 1.01 doublings/hour in the presence of 5.5% ethanol, rho*/*rpsL*^WT^ cells grew at 0.85 doublings/hour, and both *rho*^WT^/*rpsL*^WT^ and *rho*^WT^/rpsL* cells showed no or negligible growth. Conversely, in LB alone the double mutant was less fit than all other allelic combinations (despite the beneficial effects of rho* in isolation.). Thus, rho* shows a positive epistatic interactions with rpsL* in ethanol-containing media and a negative epistatic interaction in the absence of ethanol.

## Discussion

**Figure 6:**
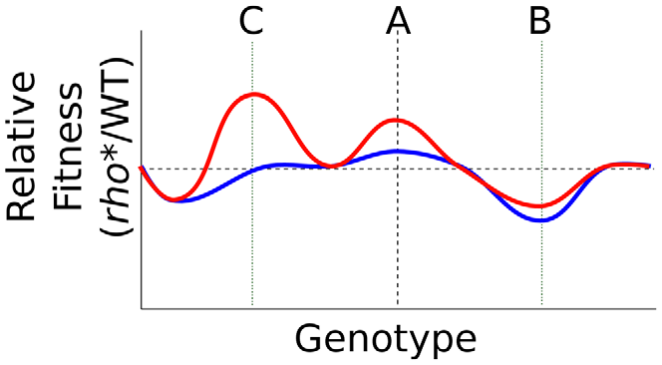
Transformation of the fitness landscape caused by rho*. Shown is a cartoon of the relative fitness of rho* vs. *rho*^WT^ cells under typical growth conditions (blue) or a stress condition (red), with genotypic variations running along the x axis. Genotype A in each case represents a wild type background (aside from the status at *rho*). rho* may directly alter fitness under the stress conditions, interact epistatically with secondary mutations (genotype B, *e.g.* Δ*visC*), and uncover further beneficial mutations under stress conditions (genotype C, e.g. rpsL*).

The wholesale reworking of the cell's fitness landscape due to rho* illustrates its potential to open evolutionary paths that would not otherwise be accessible. rho* provides both direct fitness effects and broadly varying (and often positive) epistatic relationships with perturbations at other loci, allowing it to provide benefits early in an evolutionary trajectory while at the same time providing a different, and frequently larger, profile of possible adaptive secondary mutations (see Tables S3 and S7). The interaction between rho* and rpsL* described above represents one such case: rho* itself provides a beneficial fitness effect in the presence of ethanol, and also exhibits positive epistasis with a mutation at the *rpsL* locus. A more general schematic is shown in Figure 6: the fitness effects of mutations throughout the genome are strongly influenced by the genotype at *rho* (and presumably other core transcriptional proteins as well), making some secondary mutations more or less beneficial than they would be otherwise (Figure 6, genotype B). Mutations such as rho* can also both provide a fitness benefit relative to the wild type under common growth conditions, and reveal higher fitness genotypes upon exposure to stress conditions (Figure 6, genotype C). rho* is expected to exert its effects simply by altering transcription (in this case primarily by allowing expression of regions which would not otherwise be transcribed); we thus expect that mutations to other core components of the cell's transcriptional machinery, or to other broadly influential regulators, would show similar levels of evolutionary and phenotypic leverage.

In support of this view, mutations to *rho* [10]–[13], RNA polymerase [11], [13], [20]–[23] and DNA supercoiling proteins [24]–[26] have frequently been observed in a variety of other recent directed evolution experiments. In a few cases, specific epistatic interactions involving these core transcriptional components were found to shape the future adaptive trajectory of populations. For example, Applebee and coworkers [23] found that in a set of *E. coli* populations evolved to grow efficiently in glycerol minimal media, RNA polymerase mutations arising earlier in the evolutionary trajectories showed positive epistasis with subsequent *glpK* mutations (and possibly mutations to *dapF* and *murE* as well). Similarly, in analyzing populations from an extremely long-term evolution experiment, Woods *et al.* [26] found the presence of two variant *topA* alleles in competition; of these, the allele present in the subsequently evolved strain had a less positive direct effect on fitness, but also showed positive epistasis with a secondary mutation at *spoT* that yielded an overall higher fitness phenotype. In general, these previous studies have not, however, fully explored the full breadth of both direct phenotypic and epistatic effects of the housekeeping mutations that they identified.

Because the primary effect of a hypomorphic *rho* allele such as rho* is to allow expression of regions of the genome that would not typically be expressed (see above; also [3], [4]), we thus see that the impairment of a system setting baseline boundaries for gene expression can in fact bring forth beneficial, but normally hidden, phenotypes. The concept that robustness to the effects of mutations may facilitate adaptive evolution by allowing the accumulation of genetic diversity that can be subsequently released by a single perturbation, has been proposed repeatedly in the theoretical literature. Wagner [27] discussed the “neutral space" of a biological system – a range of equivalent solutions to a given condition – and notes that the presence of diversity within the neutral space allows variation that may be useful under subsequently encountered conditions. Draghi *et al.* [28] illustrated precisely this phenomenon more quantitatively using a computational model, showing that intermediate levels of robustness (modeled as the probability of a given mutation being neutral) accelerated the adaptation of populations by providing a reservoir of phenotypically neutral genetic diversity, including variants that could be adaptive under changing conditions. More recently, in modeling tradeoffs involved in the regulation of translational readthrough, Rajon and Masel [29] found bistable solutions which required either global regulation to reduce readthrough rates, or a combination of higher readthrough rates but reduced incidence of deleterious products upon readthrough; the high readthrough rate solution was found to be more evolvable by allowing the accumulation of non-deleterious genetic diversity downstream of translational stop sites, which can subsequently be incorporated through a single mutation to the stop codon.

The behavior of rho* is also reminiscent of two phenomena related to the core translational machinery of yeast. Jarosz *et al.* [30] recently showed that the chaperone Hsp90 acts to suppress the effects of genetic variation occurring naturally between yeast strains; temperature stress or chemical inhibition of Hsp90 yielded a wide variety of phenotypic changes among ∼100 different yeast strains under 100 low-level stress conditions, frequently with differing signs of effect on fitness for different strains under the same condition. Furthermore, the authors found that Hsp90 in fact shows epistatic interactions with 20% of naturally occurring genetic variations between the strains under consideration. Similar phenomena have been observed for the yeast prion state *[PSI^+^]* [31]–[33], where (as with rho*) an alteration in the behavior of a regulatory protein gives rise to a highly pleiotropic phenotype which may be harmful or beneficial under a variety of conditions, interacts strongly with the precise genetic background of the cell in question, and appears to exert its effects by causing ectopic expression of sequences which are generally silent. The comparison between both mechanisms in yeast and rho* must not be taken too far, as there are also substantial differences, most notably in that Hsp90 and *[PSI^+^]* act post-transcriptionally, *[PSI^+^]* in particular represents an epigenetic rather than genetic mechanism, and both the prion states and Hsp90 relaxation have been shown to be encouraged by environmental stress [30], [34], whereas no similar mechanism would be expected to mutate core housekeeping genes in stressed *E. coli* cells preferentially. Nevertheless, the effects of both yeast mechanisms, and bacterial *rho* mutations, illustrate that microorganisms possess the genetic potential to grow under a broader array of conditions than their regulatory logic allows, that some of the hidden potential may be unlocked through perturbations of core regulatory proteins, and that even a single such perturbation may unleash a wide variety of positive or negative effects and interactions with other loci throughout the genome.

Taken together, our findings illustrate that a single amino acid substitution in the global transcriptional terminator Rho leads to a wholly different regulatory and phenotypic state, in which gene expression is globally altered and cellular fitness in a broad variety of environments has changed. The same mutation also dramatically alters the fitness landscape with regard to other genetic variations, making accessible a number of beneficial secondary mutations that are otherwise neutral or deleterious. The set of states reachable through rho* or other point mutations of core regulatory proteins comprise a previously underappreciated reservoir of additional phenotypes accessible to bacterial populations under selective conditions. These findings imply a role for mutations to regulators such as *rho* both as evolutionary catalysts, by making a variety of secondary mutations more favorable than they would be in the parental strain, and as evolutionary capacitors [35], by allowing silently accumulating genetic diversity to take effect rapidly upon changes in gene regulation. The full extent to which this capacity of core housekeeping and regulatory proteins is used during evolutionary trajectories, and the identity of the complete set of genes showing such broadly influential behavior, are not yet clear. It is also intriguing to speculate that classical global regulators may also show similarly diverse effects, either upon genetic perturbation or as a response to environmental signals, given that the number of genes substantially perturbed by rho* (∼200) is comparable to the number directly or indirectly affected by each global regulator (*e.g.*, CRP, IHF, or FNR) [36].

## Materials and Methods

A complete listing of strains in the present study, including abbreviations used throughout the text, is given in Table S8, and PCR primers are shown in Table S9. The *E. coli* K12 strain MG1655 [37] (ATCC strain 700926) provides the genetic background for all experiments reported here. For measurement of transcript abundances, cells were grown to mid-log phase in M9t/glucose, and RNA extracted using total RNA purification kit (Norgen Biotek, Cat 17200). After poly-A tailing, the extracted WT and rho* RNA were separately labeled, pooled, and then hybridized to Agilent custom arrays tiling the whole genome at 50 bp intervals, alternating between strands. Transposon mutagenized libraries were prepared as described by Girgis *et al.* [19]. Selections were carried out for 16 hours in 25 mL of either selective or reference media, and genomic DNA isolated using a DNeasy Blood and Tissue Kit (Qiagen). The transposon footprinting and labeling protocol for quantifying relative fitness under different conditions is described by Girgis *et al.* [18]. Bacterial growth curves were measured in Costar 96-well clear polystyrene plates, using either a Biotek Synergy MX or Powerwave XS2 plate reader (Biotek; Winooski, VT). Plates were incubated at 37°C with continuous shaking, and optical density (OD) reads at 600 nm taken every 10 minutes. Abbreviations for nutrient sources and antibiotics are given in Table S10. Complete methodological details are provided in Section 1 of Text S1, as well as Figure S9.

## Supporting Information

Figure S1 Two example loci showing over-expression in rho* (top) or WT (bottom) cells. In each case, + and − strand RNA are shown separately; the graphs show the smoothed log_2_ ratio of rho*/WT RNA at each probe.
(TIF)

Figure S2 rho* disproportionately increases antisense transcription. (A) Proportion of significant probes that are over- or under-expressed in rho* cells, subdivided by whether they are sense or antisense to the gene that they overlap (four significant probes, all overexpressed in rho*, are both sense and antisense to known genes because they overlap various *sib* loci; all four are excluded from the chart). (B) log_10_ ratios of rho* to *rho*^WT^ transcription downstream of the antisense transcription start sites identified by Dornenburg *et al.* [15]. Each point represents a 250 bp window (windows are spaced at 50 bp increments and thus overlap); the values shown are the median across all 1,005 sites of the value of the median in the appropriate window downstream of that transcription start site. A loess smoothing of the points is shown as a blue dashed line. Green and red dashed lines represent, respectively, the median and 97.5th percentile from a set of 10,000 resampled data sets calculated under random circular permutations of the transcription data; all values are offset by the median of the resampled data sets to center the distribution.
(TIF)

Figure S3 Complete iPAGE output showing the set of pathways with significant patterns of over- or under-expression in WT vs. rho* cells. Expression data were quantized with breaks corresponding to 3-, 2-, and 1.5-fold differences in either direction. At the head of each matrix the distribution of probe-level intensities present at each cluster is shown. Each matrix entry is then colored based on the significance level of over-representation (red) or underrepresentation (blue) of the corresponding cluster in probes belonging to each pathway.
(TIF)

Figure S4 Typical growth curves for rho* and Δ*yagM* strains in M9t/glu+STP (2 µg/mL).
(TIF)

Figure S5 Microarray results in the vicinity of *yagM*. Top: Relative RNA abundance between WT and rho* cells growing in M9t/glucose. The raw log_2_ ratio (WT/rho*) was smoothed separately along each strand using a Gaussian kernel with width equal to one probe (100 bp). Bottom: Z-scores from transposon library selections comparing growth in M9t/glucose+streptomycin (1.25 µg/mL) with M9t/glucose, smoothed using a running median over a 500 bp window. Negative scores indicate enrichment of an insertion in the selected condition relative to the unselected condition.
(TIF)

Figure S6 Microarray results in the vicinity of *aroM*. Top: Relative RNA abundance between WT and rho* cells growing in M9t/glucose. The raw log2 ratio (WT/rho*) was smoothed separately along each strand using a Gaussian kernel with width equal to one probe (100 bp). Bottom: Z-scores from transposon library selections comparing growth in M9t/α-keto glutarate with M9t/glucose, smoothed using a running median over a 500 bp window. Negative scores indicate enrichment of an insertion in the selected condition relative to the unselected condition.
(TIF)

Figure S7 Smoothed depletion score profile from global linkage experiments comparing growth in LB and LB+5.5% ethanol. (A) Depletion scores (i.e., ratio of transposon insertion frequency under unselective vs. selective conditions) for insertion of fragments from the tagged rho* genome into the fully evolved, ethanol tolerant strain (HGDE3) from Goodarzi *et al.* [7]. (B) Depletion scores for the evolved ethanol tolerant strain receiving DNA from the parental strain (data from Goodarzi *et al.* [7]). Genomic coordinates run clockwise starting from the indicated zero.
(TIF)

Figure S8 Typical growth rate data for rho* and rpsL* strains in LB with 5.5% ethanol added. Data points before and after three doublings of the initial optical density are shown as open and filled circles, respectively. Linear regressions are included for the rho*/*rpsL*^WT^ and rho*/rpsL* cases as dashed lines.
(TIF)

Figure S9 Growth curves in M9t/NADM media. Raw (left) and log-transformed (right) growth curves for all replicates of WT (blue) and rho* (red) cells growing in M9t/NADM on a representative day (after removal of outlier wells which appeared to show optical artifacts). Dashed lines indicate the boundaries of the region used for calculating the effective growth rates.
(TIF)

Table S1 Lag times and times to saturation for rho* vs. *rho*^WT^ cells under a variety of conditions. Antibiotic-containing conditions and carbon sources yielding extremely slow growth were omitted because they show atypical growth curves for which classical definitions of lag time do not apply (and are often negative). Ranges indicate 95% confidence intervals based on draws from the posterior distributions of model parameters. Quantities showing significant differences are bolded if they favor rho* cells over *rho*^WT^, and italicized if they favor *rho*^WT^ cells over rho* (for growth rates, we consider all values relative to growth in M9t/glucose when determining whether they favor WT or rho* cells).
(PDF)

Table S2 Comparison of growth rates for selected knockouts with those for WT or rho* cells in the MG1655 background. For each gene considered, the median RNA log_2_ ratio (rho*/*rho*^WT^) is shown for sense and antisense probes before and after the slash, respectively; the selection Z-score for all probes centered inside the target gene is also shown. Note that the RNA is isolated from WT and rho* cells, not the knockouts under consideration. Growth rate ratios Γ are defined as described in Text S1 (Eq. S4). Confidence intervals (95%) for the growth rate ratios are calculated based on 10,000 samples from the posterior distribution of model parameters. All significant quantities (defined as described in the text) are bolded. †: Growth rates calculated using spline-guided fitting.
(PDF)

Table S3 Number of distinct genes (prior to slash) and intergenic regions (after slash) containing at least one probe flagged as having a significant fitness effect in selections of transposon-mutagenized libraries. “Unique" indicates the set of genes which contained one or more significant probes in one genetic background (WT or rho*) but not the other. “Strict" indicates that the threshold for significance calling was relaxed for the background not being considered (e.g., the entry for “WT unique (strict)" contains the number of genes which had significant probes using the strict criteria in the WT selection, but no significant probes using relaxed criteria in the corresponding rho* selection); see Section 1.5 of Text S1 for details.
(PDF)

Table S4 Growth rates associated with secondary mutations (labeled A) found to interact non-multiplicatively with rho*. Growth rates γ are given in doublings/hour; the absolute epistasis ε is calculated using Eq. S7 (see Text S1), with a 95% confidence interval obtained via resampling of the posterior distribution of model parameters. Concentrations of ethanol, CML (chloramphenicol), and STP (streptomycin) were 5.5% (v/v), 1.875 µg/mL, and 2.0 µg/mL, respectively. †: Growth rates calculated using spline-based fitting; see Section 1 of Text S1 for details. ‡: Relative fitnesses obtained from competition experiments (see Text S1, Section 1.9) thus, growth rates are omitted.
(PDF)

Table S5 Results of direct competition experiments used to provide an additional test for epistatic interactions identified from growth rate data. Values of the multiplicative epistasis are shown based on growth rate data and on competition experiments. “p(|ε|)" refers to the posterior probability (from competition experiments) that the epistasis ε is of the same sign as identified from growth curve data.
(PDF)

Table S6 Growth rates obtained from replicate experiments and pooled data on growth of rho*/*rpsL*^WT^ and rho*/rpsL* cells in LB+5.5% ethanol. In each case a linear regression was used on data from log-transformed growth of exponential-phase cells, as described in Text S1. “Combined" indicates the results of fitting a linear mixed-effects model to the full data set.
(PDF)

Table S7 Comparison of evolvability of *rho*^WT^ and rho* cells based on transposon-mutagenized library selections. The last four columns show the number of distinct genes containing at least one probe flagged as significant which gave an advantage (adv.) or disadvantage (dis.) to cells carrying the corresponding insertion during selection experiments in transposon-mutagenized libraries. For comparison, growth rates of both strains are given in doublings per hour; all differences between growth rates under reference and selective conditions were significant (no overlap in 95% confidence intervals) except for the rho*/STP case. Of note, rho* cells show greater evolvability (in terms of the number of available adaptive secondary mutations) under three of the four conditions, and under the fourth (STP) the amount of antibiotic used relative to the tolerance of the rho* cells is so low that little room for improvement is even present.
(PDF)

Table S8 Strains used in the current study. The entry in the “Name" column is used to refer to a given strain throughout the text.
(PDF)

Table S9 Primer pairs used over the course of the current study.
(PDF)

Table S10 Abbreviations for nutrient sources and antibiotics used throughout the study.
(PDF)

Table S11 Number of technical replicates used to calculate growth rates and other statistics for each strain/growth condition combination. One well on a 96 well plate is considered one replicate. Technical replicates for each entry were in all cases spread across at least two different plate reader runs on different days. The tabulated counts do not include pruned replicates; see Text S1 for details. †: replicates used for spline-based fitting. ‡: replicates used for effective growth rate.
(PDF)

Text S1 Detailed methods, extended discussion, and supplementary results.
(PDF)
