# Correction: Fitness Landscape Transformation through a Single Amino Acid Change in the Rho Terminator

**DOI:** 10.1371/journal.pgen.1004940

**Published:** 2015-03-05

**Authors:** The PLOS Genetics Staff

In the Results section, the rpsL* mutation is erroneously stated to be a nonsense mutation, but is in fact a missense mutation (R54S). The genomic location and identity of the mutation listed in the supplementary material remains correct (n.b. sequence numbering refers to the U00096.2 reference sequence).
