# Supplementary figures and images for: Fitness Landscape Transformation through a Single Amino Acid Change in the Rho Terminator
Source: PLoS Genet. 2012 May 31;8(5):e1002744. doi: 10.1371/journal.pgen.1002744 (PMC3364947; doi:10.1371/journal.pgen.1002744)

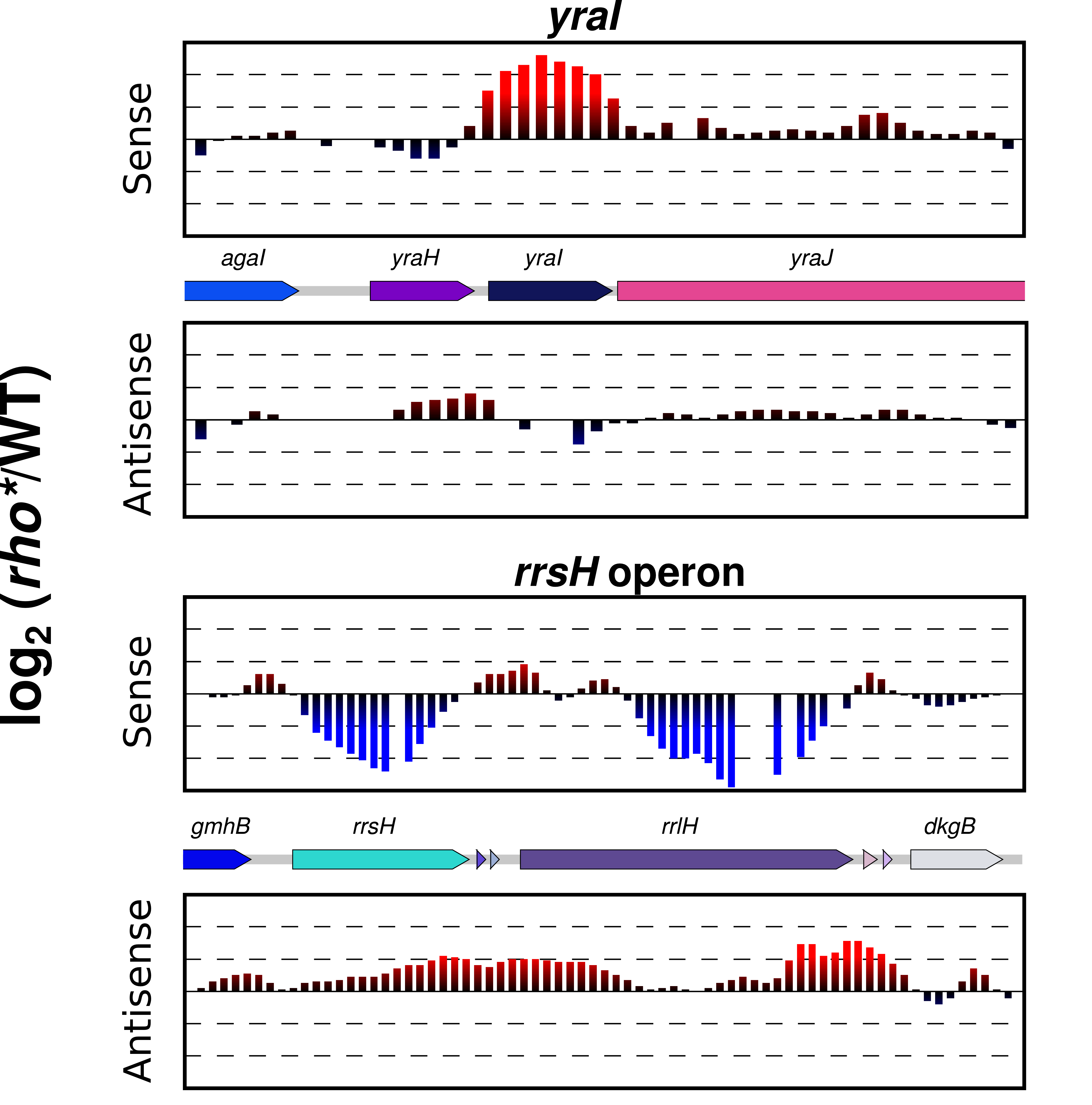

Supplement: Figure S1 — Two example loci showing over-expression in rho* (top) or WT (bottom) cells. In each case, + and − strand RNA are shown separately; the graphs show the smoothed log2 ratio of rho*/WT RNA at each probe. (TIF) [file pgen.1002744.s001.tif]

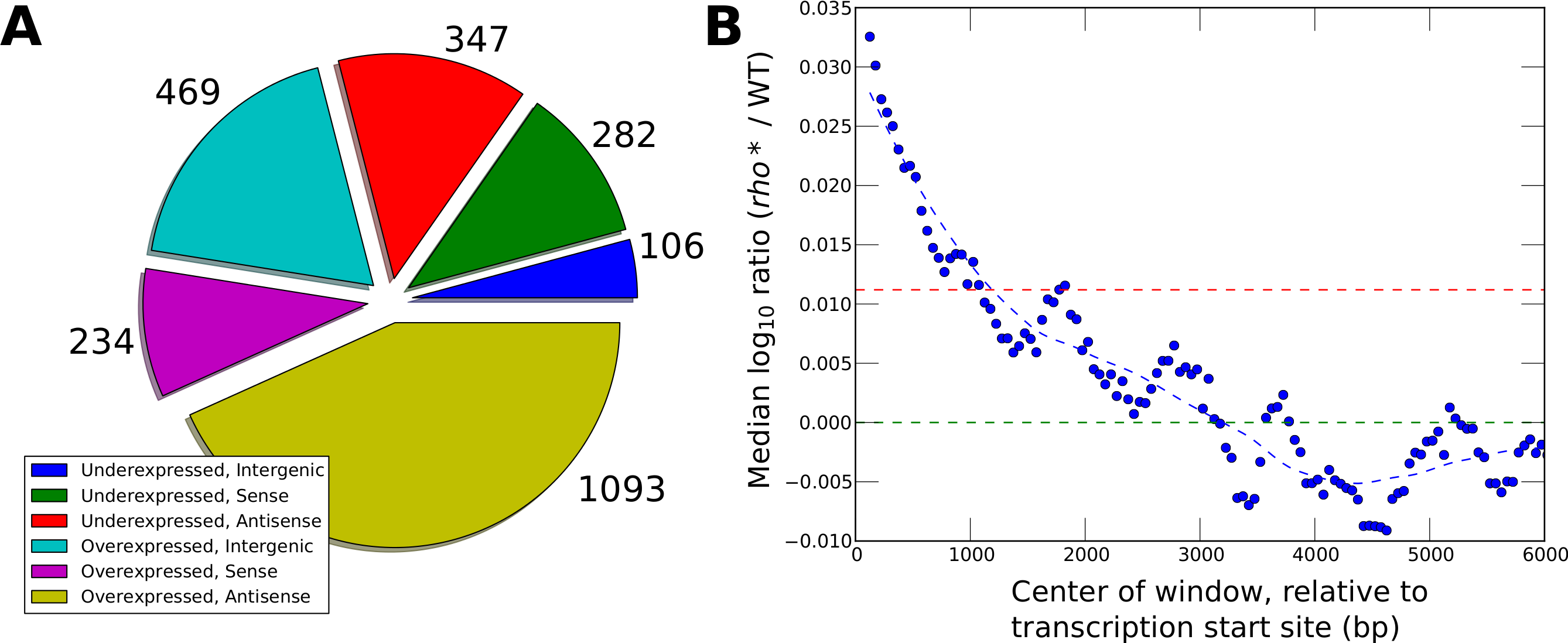

Supplement: Figure S2 — rho* disproportionately increases antisense transcription. (A) Proportion of significant probes that are over- or under-expressed in rho* cells, subdivided by whether they are sense or antisense to the gene that they overlap (four significant probes, all overexpressed in rho*, are both sense and antisense to known genes because they overlap various sib loci; all four are excluded from the chart). (B) log10 ratios of rho* to rhoWT transcription downstream of the antisense transcription start sites identified by Dornenburg et al. [15]. Each point represents a 250 bp window (windows are spaced at 50 bp increments and thus overlap); the values shown are the median across all 1,005 sites of the value of the median in the appropriate window downstream of that transcription start site. A loess smoothing of the points is shown as a blue dashed line. Green and red dashed lines represent, respectively, the median and 97.5th percentile from a set of 10,000 resampled data sets calculated under random circular permutations of the transcription data; all values are offset by the median of the resampled data sets to center the distribution. (TIF) [file pgen.1002744.s002.tif]

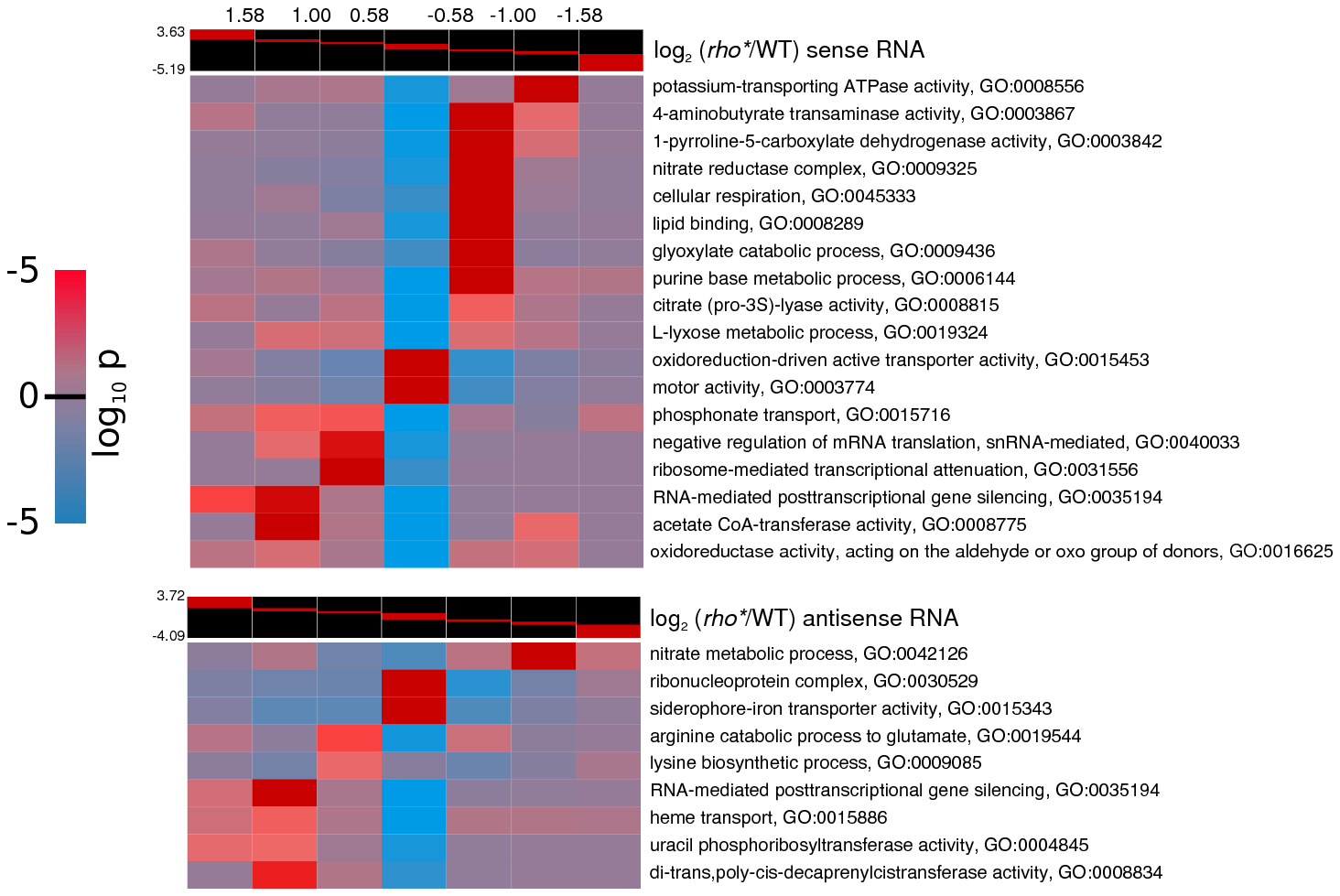

Supplement: Figure S3 — Complete iPAGE output showing the set of pathways with significant patterns of over- or under-expression in WT vs. rho* cells. Expression data were quantized with breaks corresponding to 3-, 2-, and 1.5-fold differences in either direction. At the head of each matrix the distribution of probe-level intensities present at each cluster is shown. Each matrix entry is then colored based on the significance level of over-representation (red) or underrepresentation (blue) of the corresponding cluster in probes belonging to each pathway. (TIF) [file pgen.1002744.s003.tif]

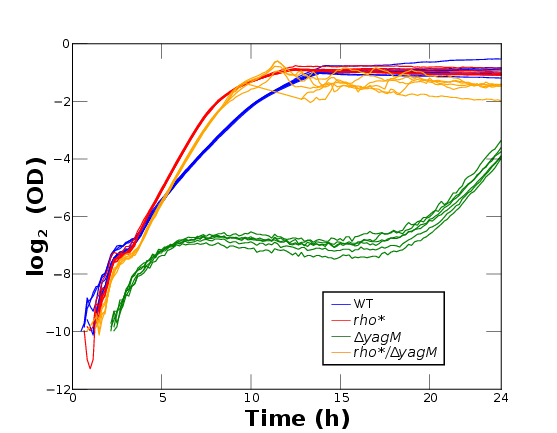

Supplement: Figure S4 — Typical growth curves for rho* and ΔyagM strains in M9t/glu+STP (2 µg/mL). (TIF) [file pgen.1002744.s004.tif]

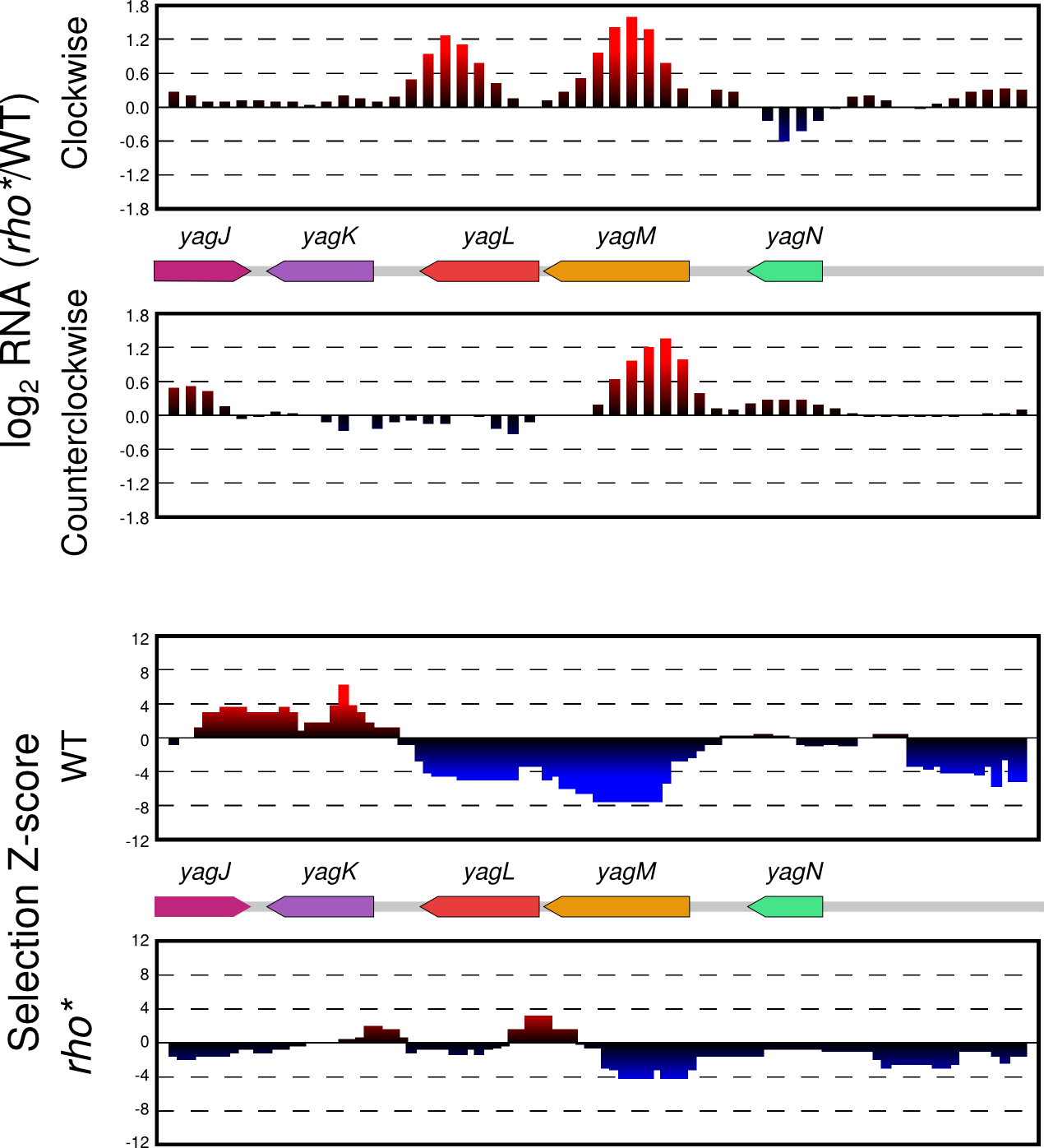

Supplement: Figure S5 — Microarray results in the vicinity of yagM. Top: Relative RNA abundance between WT and rho* cells growing in M9t/glucose. The raw log2 ratio (WT/rho*) was smoothed separately along each strand using a Gaussian kernel with width equal to one probe (100 bp). Bottom: Z-scores from transposon library selections comparing growth in M9t/glucose+streptomycin (1.25 µg/mL) with M9t/glucose, smoothed using a running median over a 500 bp window. Negative scores indicate enrichment of an insertion in the selected condition relative to the unselected condition. (TIF) [file pgen.1002744.s005.tif]

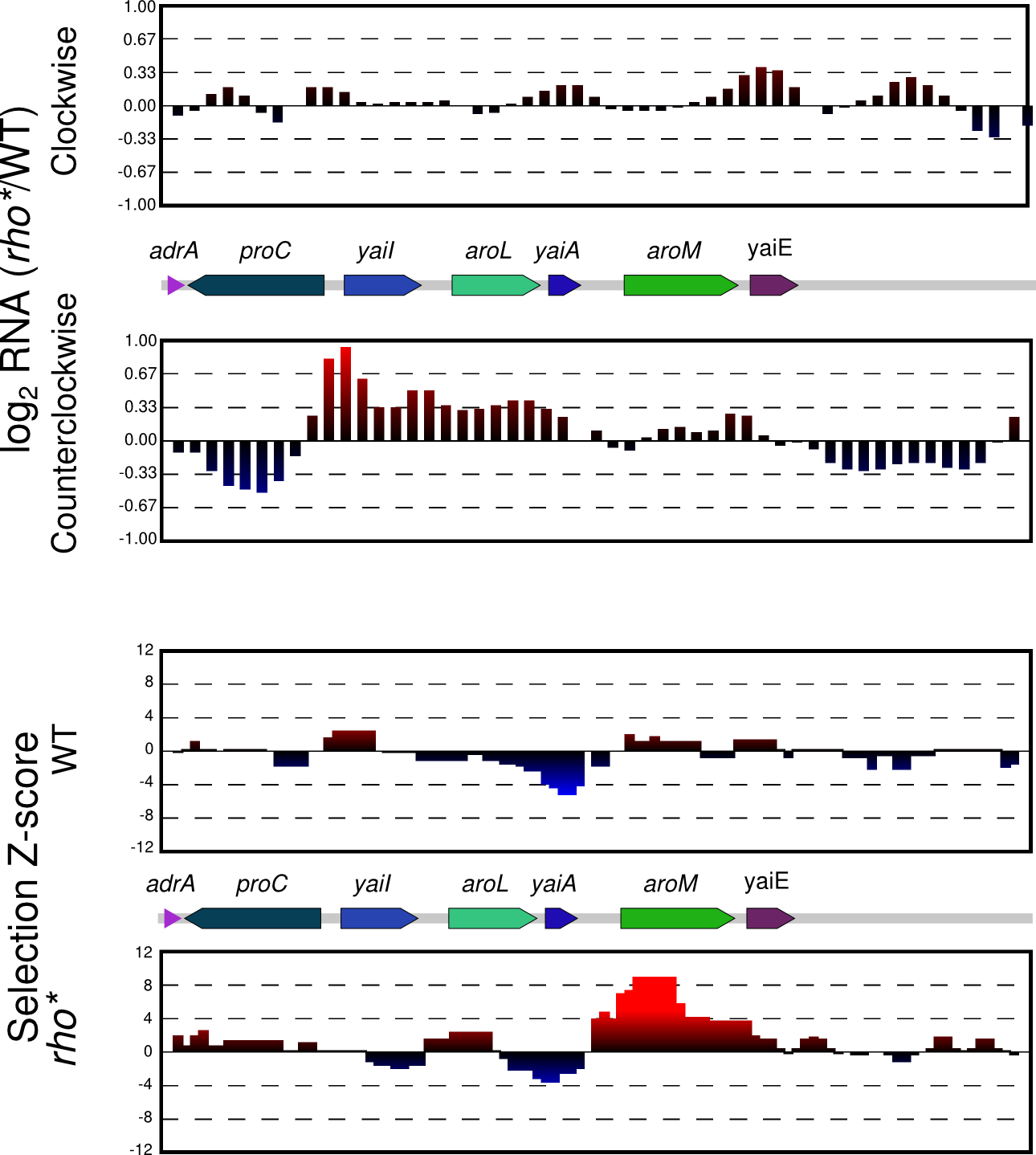

Supplement: Figure S6 — Microarray results in the vicinity of aroM. Top: Relative RNA abundance between WT and rho* cells growing in M9t/glucose. The raw log2 ratio (WT/rho*) was smoothed separately along each strand using a Gaussian kernel with width equal to one probe (100 bp). Bottom: Z-scores from transposon library selections comparing growth in M9t/α-keto glutarate with M9t/glucose, smoothed using a running median over a 500 bp window. Negative scores indicate enrichment of an insertion in the selected condition relative to the unselected condition. (TIF) [file pgen.1002744.s006.tif]

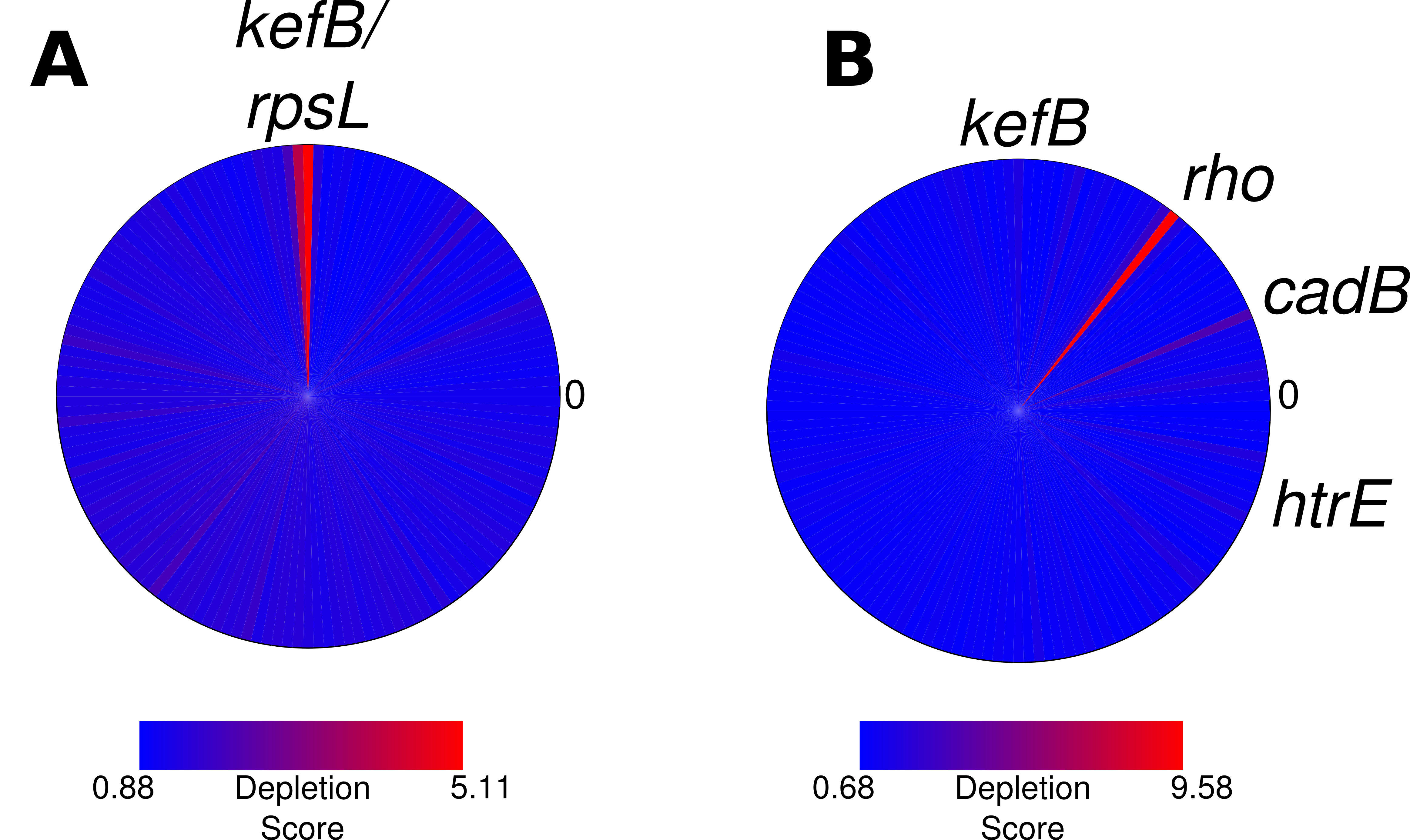

Supplement: Figure S7 — Smoothed depletion score profile from global linkage experiments comparing growth in LB and LB+5.5% ethanol. (A) Depletion scores (i.e., ratio of transposon insertion frequency under unselective vs. selective conditions) for insertion of fragments from the tagged rho* genome into the fully evolved, ethanol tolerant strain (HGDE3) from Goodarzi et al. [7]. (B) Depletion scores for the evolved ethanol tolerant strain receiving DNA from the parental strain (data from Goodarzi et al. [7]). Genomic coordinates run clockwise starting from the indicated zero. (TIF) [file pgen.1002744.s007.tif]

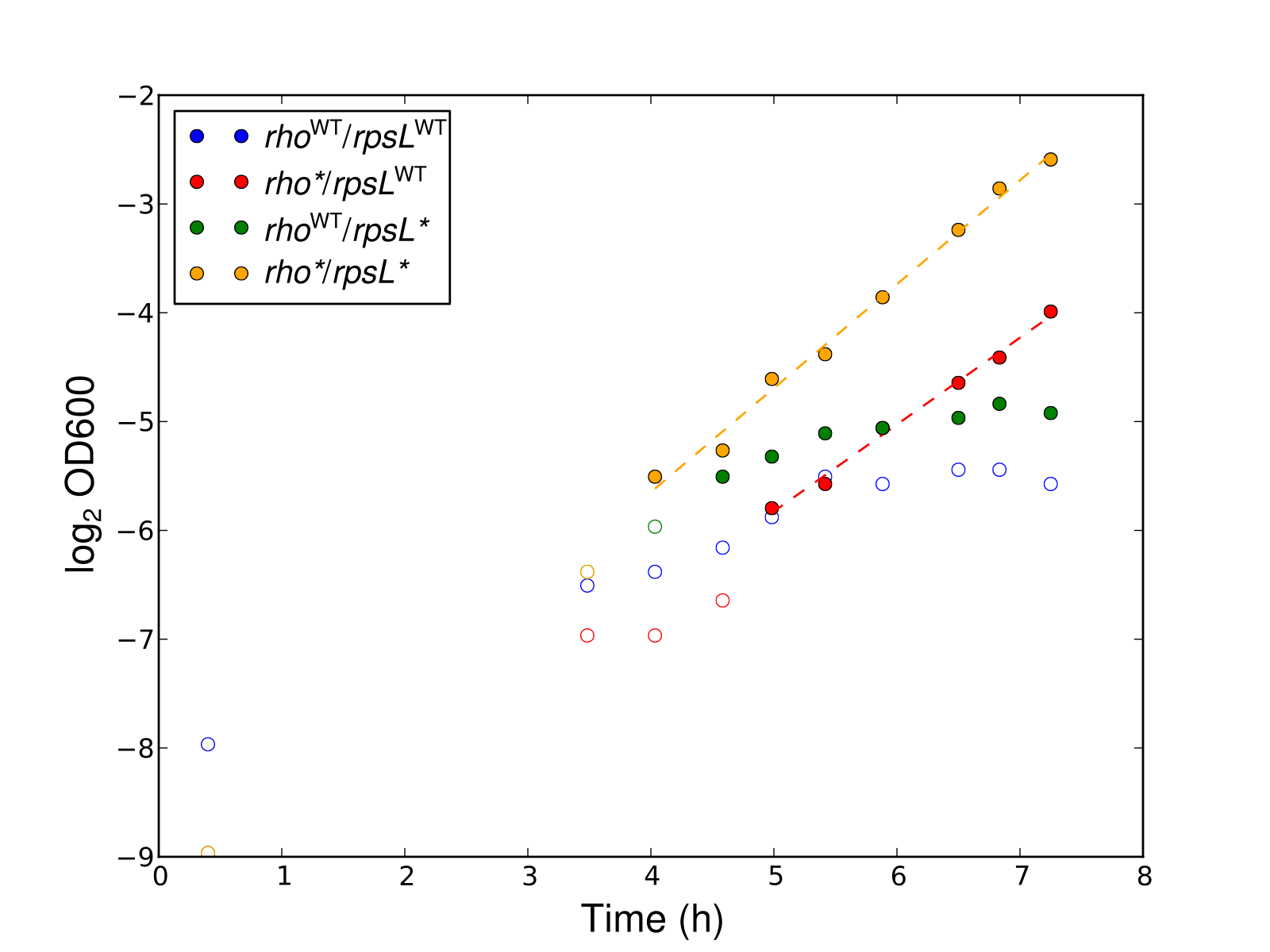

Supplement: Figure S8 — Typical growth rate data for rho* and rpsL* strains in LB with 5.5% ethanol added. Data points before and after three doublings of the initial optical density are shown as open and filled circles, respectively. Linear regressions are included for the rho*/rpsLWT and rho*/rpsL* cases as dashed lines. (TIF) [file pgen.1002744.s008.tif]

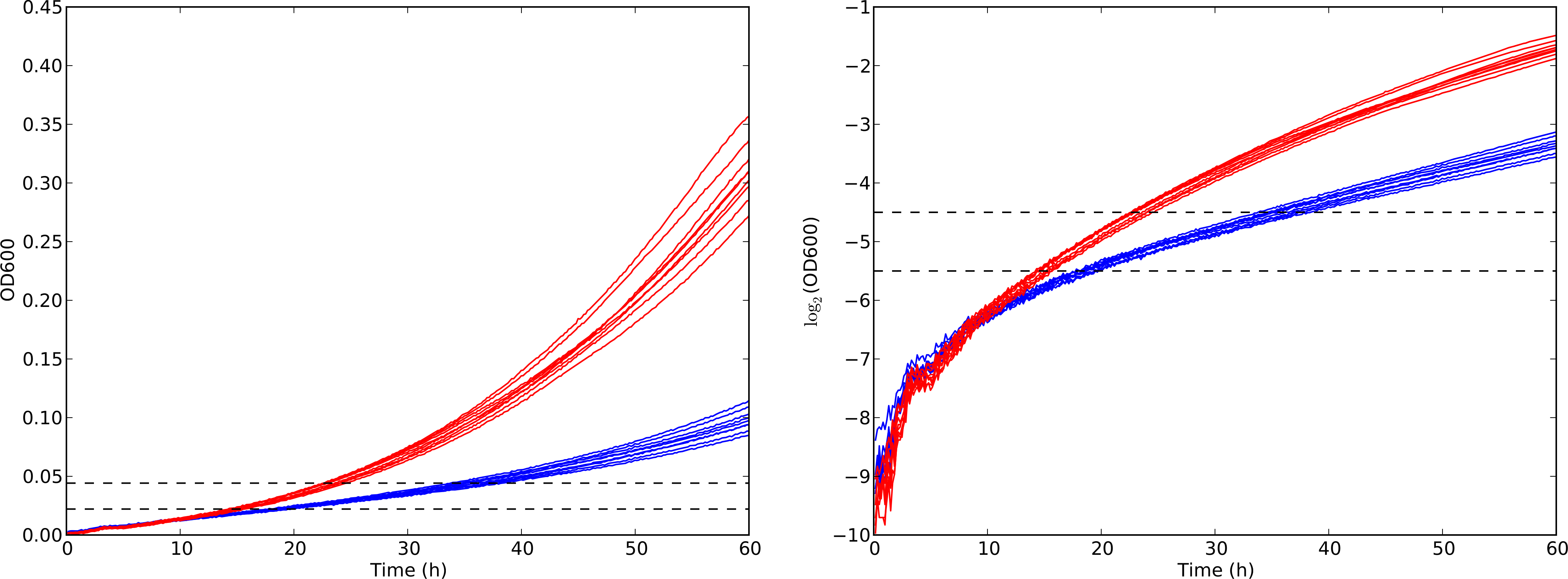

Supplement: Figure S9 — Growth curves in M9t/NADM media. Raw (left) and log-transformed (right) growth curves for all replicates of WT (blue) and rho* (red) cells growing in M9t/NADM on a representative day (after removal of outlier wells which appeared to show optical artifacts). Dashed lines indicate the boundaries of the region used for calculating the effective growth rates. (TIF) [file pgen.1002744.s009.tif]
