# Supplementary material for: Fitness Landscape Transformation through a Single Amino Acid Change in the Rho Terminator
Source: PLoS Genet. 2012 May 31;8(5):e1002744. doi: 10.1371/journal.pgen.1002744 (PMC3364947; doi:10.1371/journal.pgen.1002744)
Supplement: Table S1 — Lag times and times to saturation for rho* vs. rhoWT cells under a variety of conditions. Antibiotic-containing conditions and carbon sources yielding extremely slow growth were omitted because they show atypical growth curves for which classical definitions of lag time do not apply (and are often negative). Ranges indicate 95% confidence intervals based on draws from the posterior distributions of model parameters. Quantities showing significant differences are bolded if they favor rho* cells over rhoWT, and italicized if they favor rhoWT cells over rho* (for growth rates, we consider all values relative to growth in M9t/glucose when determining whether they favor WT or rho* cells). (PDF) [file pgen.1002744.s010.pdf]

Table S1: Lag times and times to saturation for *rho** vs. rho^{WT} cells under a variety of conditions. Antibiotic-containing conditions and carbon sources yielding extremely slow growth were omitted because they show atypical growth curves for which classical definitions of lag time do not apply (and are often negative). Ranges indicate 95% confidence intervals based on draws from the posterior distributions of model parameters. Quantities showing significant differences are bolded if they favor *rho** cells over rho^{WT}, and italicized if they favor rho^{WT} cells over *rho** (for growth rates, we consider all values relative to growth in M9t/glucose when determining whether they favor WT or *rho** cells).

**Media**	**Ratio of growth rates (rho*/ rho^{WT})**	**Difference of lag times (rho*- rho^{WT})**	**Difference of saturation times (rho*- rho^{WT})**
M9t/acetate	**1.171**	**-4.902 (-8.777 – -1.001)**	-1.627 (-6.330–3.049)
M9t/AKG	*1.031*	*1.729 (0.941 – 2.519)*	0.177 (-0.760 – 1.121)
M9t/arabinose	**1.092**	-0.457 (-0.990 – 0.056)	5.351 (-13.436 – 25.248)
M9t/glucose	1.061	-0.009 (-0.137 – 0.117)	-0.008 (-2.103 – 2.125)
M9t/glycerol	**1.102**	-0.023 (-0.538 – 0.491)	**-2.850 (-5.405 – -0.328)**
M9t/lactose	**1.133**	-0.284 (-0.647 – 0.083)	**-6.165 (-10.479 – -2.462)**
LB	1.052	*0.229 (0.063 – 0.400)*	**-3.230 (-5.012 – -1.369)**
M9t/pyruvate	*0.879*	0.645 (-0.645 – 1.954)	-0.455 (-3.564 – 2.716)
M9t/Tween20	*0.947*	**-5.456 (-10.563 – -0.140)**	4.205 (-1.696 – 10.306)
M9t/xylose	**1.179**	-0.087 (-0.362 – 0.185)	-8.131 (-22.546 – 6.256)
M9t/ribose	**1.155**	**-3.693 (-5.951 – -1.483)**	-1.357 (-3.460 – 0.977)
M9t/lactate	*0.930*	*1.658 (1.294 – 2.020)*	3.287 (-0.667 – 7.232)
