# Supplementary material for: Fitness Landscape Transformation through a Single Amino Acid Change in the Rho Terminator
Source: PLoS Genet. 2012 May 31;8(5):e1002744. doi: 10.1371/journal.pgen.1002744 (PMC3364947; doi:10.1371/journal.pgen.1002744)
Supplement: Table S2 — Comparison of growth rates for selected knockouts with those for WT or rho* cells in the MG1655 background. For each gene considered, the median RNA log2 ratio (rho*/rhoWT) is shown for sense and antisense probes before and after the slash, respectively; the selection Z-score for all probes centered inside the target gene is also shown. Note that the RNA is isolated from WT and rho* cells, not the knockouts under consideration. Growth rate ratios Γ are defined as described in Text S1 (Eq. S4). Confidence intervals (95%) for the growth rate ratios are calculated based on 10,000 samples from the posterior distribution of model parameters. All significant quantities (defined as described in the text) are bolded. †: Growth rates calculated using spline-guided fitting. (PDF) [file pgen.1002744.s011.pdf]

Table S2: Comparison of growth rates for selected knockouts with those for WT or *rho** cells in the MG1655 background. For each gene considered, the median RNA $\log_2$ ratio (*rho** / rho^{WT}) is shown for sense and antisense probes before and after the slash, respectively; the selection Z-score for all probes centered inside the target gene is also shown. Note that the RNA is isolated from WT and *rho** cells, not the knockouts under consideration. Growth rate ratios Γ are defined as described in the text. Confidence intervals (95%) for the growth rate ratios are calculated based on 10,000 samples from the posterior distribution of model parameters. All significant quantities (defined as described in the text) are bolded. †: Growth rates calculated using spline-guided fitting.

Strain	Compound	RNA $\log_2$ ratio	WT selection Z-score	*rho** selection Z-score	WT Γ (95% CI)	*rho** Γ (95% CI)
Δ *yagM*	STP (2.0 μg/mL)	-0.501 / -0.707	**-7.423**	-0.589	**0.532† (0.476–0.592)**	0.953† (0.880 – 1.031)
Δ *ykgL*		-0.232 / -0.669	**10.646**	-1.408	**0.890† (0.827–0.958)**	1.001† (0.934 – 1.071)
Δ *envZ*	CML (1.875 μg/mL)	0.316 / 0.111	**-5.010**	**-5.758**	**1.554 (1.471 – 1.618)**	**1.537 (1.454 – 1.597)**
Δ *yadM*		-0.558 / -0.101	1.509	**12.886**	1.010 (0.947 – 1.052)	1.020 (0.957 – 1.058)
Δ *sthA*	M9t/AKG	0.283 / 0.079	-0.857	**-7.118**	0.977 (0.945 – 1.005)	**0.946 (0.913 – 0.973)**
Δ *aroM*		0.032 / -0.082	1.295	**9.086**	0.982† (0.948–1.017)	**0.907† (0.880–0.936)**
Δ *ppdD*	M9t/NADM	0.455 / 0.213	0.100	**6.846**	**1.122 (1.056–1.191)**	**0.870 (0.802–0.945)**
Δ *yadN*		-0.111 / -0.600	2.409	**-11.071**	0.959 (0.901–1.021)	**0.863 (0.796–0.937)**
