# Supplementary material for: Fitness Landscape Transformation through a Single Amino Acid Change in the Rho Terminator
Source: PLoS Genet. 2012 May 31;8(5):e1002744. doi: 10.1371/journal.pgen.1002744 (PMC3364947; doi:10.1371/journal.pgen.1002744)
Supplement: Table S3 — Number of distinct genes (prior to slash) and intergenic regions (after slash) containing at least one probe flagged as having a significant fitness effect in selections of transposon-mutagenized libraries. “Unique" indicates the set of genes which contained one or more significant probes in one genetic background (WT or rho*) but not the other. “Strict" indicates that the threshold for significance calling was relaxed for the background not being considered (e.g., the entry for “WT unique (strict)" contains the number of genes which had significant probes using the strict criteria in the WT selection, but no significant probes using relaxed criteria in the corresponding rho* selection); see Section 1.5 of Text S1 for details. (PDF) [file pgen.1002744.s012.pdf]

Table S3: Number of distinct genes (prior to slash) and intergenic regions (after slash) containing at least one probe flagged as having a significant fitness effect in selections of transposon-mutagenized libraries. "Unique" indicates the set of genes which contained one or more significant probes in one genetic background (WT or *rho**) but not the other. "Strict" indicates that the threshold for significance calling was relaxed for the background not being considered (*e.g.*, the entry for "WT unique (strict)" contains the number of genes which had significant probes using the strict criteria in the WT selection, but no significant probes using relaxed criteria in the corresponding *rho** selection); see Section 1.5 for details.

**Condition**	**WT**	**WT unique**	**WT unique (strict)**	***rho****	***rho** unique**	***rho** unique (strict)**
CML	202 / 32	139 / 27	40 / 16	380 / 65	317 / 60	155 / 42
STP	239 / 48	220 / 45	144 / 32	148 / 19	129 / 16	62 / 11
AKG	341 / 77	235 / 63	81 / 39	347 / 64	241 /50	88 /22
NADM	511 / 164	309 / 89	61 / 37	464 / 188	262 / 113	33 / 32
