# Supplementary material for: Fitness Landscape Transformation through a Single Amino Acid Change in the Rho Terminator
Source: PLoS Genet. 2012 May 31;8(5):e1002744. doi: 10.1371/journal.pgen.1002744 (PMC3364947; doi:10.1371/journal.pgen.1002744)
Supplement: Table S4 — Growth rates associated with secondary mutations (labeled A) found to interact non-multiplicatively with rho*. Growth rates γ are given in doublings/hour; the absolute epistasis ε is calculated using Eq. S7 (see Text S1), with a 95% confidence interval obtained via resampling of the posterior distribution of model parameters. Concentrations of ethanol, CML (chloramphenicol), and STP (streptomycin) were 5.5% (v/v), 1.875 µg/mL, and 2.0 µg/mL, respectively. †: Growth rates calculated using spline-based fitting; see Section 1 of Text S1 for details. ‡: Relative fitnesses obtained from competition experiments (see Text S1, Section 1.9) thus, growth rates are omitted. (PDF) [file pgen.1002744.s013.pdf]

Table S4: Growth rates associated with secondary mutations (labeled A) found to interact non-multiplicatively with *rho**. Growth rates γ are given in doublings/hour; the absolute epistasis ϵ is calculated using Eq. S7, with a 95% confidence interval obtained via resampling of the posterior distribution of model parameters. Concentrations of ethanol, CML (chloramphenicol), and STP (streptomycin) were 5.5% (v/v), 1.875 μg/mL, and 2.0 μg/mL, respectively. †: Growth rates calculated using spline-based fitting; see Section 1 for details. ‡: Relative fitnesses obtained from competition experiments (see Section 1.9) thus, growth rates are omitted.

**Media**	Secondary mutation (A)	γ_{WT}	γ_{rho*}	γ_A	$\gamma_{A,rho*}$	$\epsilon_{A,rho*}$ (95% CI)
LB+ethanol	*rpsL**	(see text)				
LB	*rpsL**	2.510	2.640	2.437	2.273	**-0.116 (-0.190 – -0.044)**
M9t/glucose†	Δ*visC*	0.951	1.018	0.828	0.704	**-0.192 (-0.224 – -0.159)**
M9t/αKG	Δ*sthA*	0.509	0.525	0.499	0.494	**-0.041 (-0.073 – -0.009)**
M9t/αKG†	Δ*aroM*	0.517	0.527	0.505	0.469	**-0.090 (-0.124 – -0.058)**
M9t/αKG	Δ*yaaI*	0.509	0.525	0.516	0.530	-0.003 (-0.039 – 0.032)
M9t/αKG	Δ*ybaM*	0.509	0.525	0.483	0.492	-0.012 (-0.046 – 0.021)
M9t/glucose+CML	Δ*envZ*	0.379	0.294	0.601	0.455	-0.030 (-0.106 – 0.041)
M9t/glucose+CML	Δ*yadM*	0.379	0.294	0.373	0.296	0.016 (-0.034 – 0.065)
M9t/glucose+CML	Δ*iraP*	0.379	0.294	0.376	0.270	**-0.056 (-0.105 – -0.009)**
M9t/glucose+CML	Δ*apaH*	0.379	0.294	0.411	0.321	0.007 (-0.050 – 0.062)
M9t/glucose+STP†	Δ*yagM*	0.660	0.960	0.355	0.924	**0.618 (0.495 – 0.737)**
M9t/glucose+STP†	Δ*ykgL*	0.660	0.960	0.581	0.948	**0.156 (0.025 – 0.277)**
M9t/NADM‡	Δ*ppdD*	–				**-0.359 (-0.518 – -0.212)**
M9t/NADM‡	Δ*yadN*	–				**-0.137 (-0.276 – 0.006)**
