# Supplementary material for: Fitness Landscape Transformation through a Single Amino Acid Change in the Rho Terminator
Source: PLoS Genet. 2012 May 31;8(5):e1002744. doi: 10.1371/journal.pgen.1002744 (PMC3364947; doi:10.1371/journal.pgen.1002744)
Supplement: Table S5 — Results of direct competition experiments used to provide an additional test for epistatic interactions identified from growth rate data. Values of the multiplicative epistasis are shown based on growth rate data and on competition experiments. “p(|ε|)" refers to the posterior probability (from competition experiments) that the epistasis ε is of the same sign as identified from growth curve data. (PDF) [file pgen.1002744.s014.pdf]

Table S5: Results of direct competition experiments used to provide an additional test for epistatic interactions identified from growth rate data. Values of the multiplicative epistasis are shown based on growth rate data and on competition experiments. "p($|\epsilon|$)" refers to the posterior probability (from competition experiments) that the epistasis ϵ is of the same sign as identified from growth curve data.

**Interaction**	**Media**	ϵ **(growth rates)**	ϵ **(selections)**	p($\|\epsilon\|$)
*rho**– Δ*visC*	M9t/glu	-0.192	-0.174	> 0.999
*rho**– *rpsL**	LB	-0.116	-0.553	0.972
*rho**– Δ*aroM*	M9t/AKG	-0.090	-0.056	0.842
*rho**– Δ*yagM*	M9t/glu+STP	0.618	-0.038	0.257
