# Supplementary material for: Fitness Landscape Transformation through a Single Amino Acid Change in the Rho Terminator
Source: PLoS Genet. 2012 May 31;8(5):e1002744. doi: 10.1371/journal.pgen.1002744 (PMC3364947; doi:10.1371/journal.pgen.1002744)
Supplement: Table S6 — Growth rates obtained from replicate experiments and pooled data on growth of rho*/rpsLWT and rho*/rpsL* cells in LB+5.5% ethanol. In each case a linear regression was used on data from log-transformed growth of exponential-phase cells, as described in Text S1. “Combined" indicates the results of fitting a linear mixed-effects model to the full data set. (PDF) [file pgen.1002744.s015.pdf]

Table S6: Growth rates obtained from replicate experiments and pooled data on growth of *rho**/*rpsL*^{WT} and *rho**/*rpsL** cells in LB+5.5% ethanol. In each case a linear regression was used on data from log-transformed growth of exponential-phase cells, as described in the text. "Combined" indicates the results of fitting a linear mixed-effects model to the full data set.

**Replicate**	*rho** **growth rate (r^2; std. error)**	*rho** /*rpsL** **growth rate (r^2, std. error)**
1	0.896 (0.995; 0.063)	1.068 (0.987; 0.062)
2	0.924 (0.990; 0.041)	1.056 (0.992; 0.040)
3	0.754 (0.999; 0.001)	0.903 (0.991; 0.034)
4	0.798 (0.993; 0.033)	0.991 (0.992, 0.033)
Combined	0.850 (n/a; 0.042)	1.006 (n/a; 0.041)
