# Supplementary material for: Fitness Landscape Transformation through a Single Amino Acid Change in the Rho Terminator
Source: PLoS Genet. 2012 May 31;8(5):e1002744. doi: 10.1371/journal.pgen.1002744 (PMC3364947; doi:10.1371/journal.pgen.1002744)
Supplement: Table S7 — Comparison of evolvability of rhoWT and rho* cells based on transposon-mutagenized library selections. The last four columns show the number of distinct genes containing at least one probe flagged as significant which gave an advantage (adv.) or disadvantage (dis.) to cells carrying the corresponding insertion during selection experiments in transposon-mutagenized libraries. For comparison, growth rates of both strains are given in doublings per hour; all differences between growth rates under reference and selective conditions were significant (no overlap in 95% confidence intervals) except for the rho*/STP case. Of note, rho* cells show greater evolvability (in terms of the number of available adaptive secondary mutations) under three of the four conditions, and under the fourth (STP) the amount of antibiotic used relative to the tolerance of the rho* cells is so low that little room for improvement is even present. (PDF) [file pgen.1002744.s016.pdf]

Table S7: Comparison of evolvability of rho^{WT} and *rho** cells based on transposon-mutagenized library selections. The last four columns show the number of distinct genes containing at least one probe flagged as significant which gave an advantage (adv.) or disadvantage (dis.) to cells carrying the corresponding insertion during selection experiments in transposon-mutagenized libraries. For comparison, growth rates of both strains are given in doublings per hour; all differences between growth rates under reference and selective conditions were significant (no overlap in 95% confidence intervals) except for the *rho**/STP case. Of note, *rho** cells show greater evolvability (in terms of the number of available adaptive secondary mutations) under three of the four conditions, and under the fourth (STP) the amount of antibiotic used relative to the tolerance of the *rho** cells is so low that little room for improvement is even present.

**Condition**	**WT growth rate**	***rho** growth rate**	**WT adv.**	**WT dis.**	***rho** adv.**	***rho** dis.**
(reference)	1.017	1.079	(n/a)			
CML	0.379	0.294	106	99	211	179
STP	0.770	1.030	144	99	61	91
AKG	0.509	0.521	152	195	159	193
NADM	0.049	0.094	217	302	260	210
