# Supplementary material for: Fitness Landscape Transformation through a Single Amino Acid Change in the Rho Terminator
Source: PLoS Genet. 2012 May 31;8(5):e1002744. doi: 10.1371/journal.pgen.1002744 (PMC3364947; doi:10.1371/journal.pgen.1002744)
Supplement: Table S8 — Strains used in the current study. The entry in the “Name" column is used to refer to a given strain throughout the text. (PDF) [file pgen.1002744.s017.pdf]

Table S8: Strains used in the current study. The entry in the "Name" column is used to refer to a given strain throughout the text.

**Name**	**Genotype**	**Source**
WT (*E. coli* MG1655)	F- λ- *ilvG*− $rfb-50$ $rph-1$	ATCC 700926 [3]
HGDE3	(see SRA045739.1)	Ethanol tolerant strain from [6]
*rho**	MG1655 with Rho F62L mutation (*rho∗*) from HGDE3	Present work
lacZ-	MG1655 *lacZ::(cat gfp)*	[6]
WT/Δ*visC*	MG1655 Δ*visC*	Present work
WT/Δ*envZ*	MG1655 Δ*envZ*	Present work
WT/Δ*yadM*	MG1655 Δ*yadM*	Present work
WT/Δ*yagM*	MG1655 Δ*yagM*	Present work
WT/Δ*ykgL*	MG1655 Δ*ykgL*	Present work
WT/Δ*ppdD*	MG1655 Δ*ppdD*	Present work
WT/Δ*yadN*	MG1655 Δ*yadN*	Present work
WT/Δ*sthA*	MG1655 Δ*sthA*	Present work
WT/Δ*aroM*	MG1655 Δ*aroM*	Present work
WT/Δ*visC*	MG1655 Δ*visC*	Present work
WT/Δ*apaH*	MG1655 Δ*apaH*	Present work
WT/Δ*ynbE*	MG1655 Δ*ynbE*	Present work
WT/Δ*yaaI*	MG1655 Δ*yaaI*	Present work
WT/Δ*ybaM*	MG1655 Δ*ybaM*	Present work
WT/*rpsL∗*	MG1655 with *rpsL* nonsense mutation (*rpsL∗*)	Present work
*rho**/Δ*visC*	MG1655 *rho** Δ*visC*	Present work
*rho**/Δ*envZ*	MG1655 *rho** Δ*envZ*	Present work
*rho**/Δ*yadM*	MG1655 *rho** Δ*yadM*	Present work
*rho**/Δ*yagM*	MG1655 *rho** Δ*yagM*	Present work
*rho**/Δ*ykgL*	MG1655 *rho** Δ*ykgL*	Present work
*rho**/Δ*ppdD*	MG1655 *rho** Δ*ppdD*	Present work
*rho**/Δ*yadN*	MG1655 *rho** Δ*yadN*	Present work
*rho**/Δ*sthA*	MG1655 *rho** Δ*sthA*	Present work
*rho**/Δ*aroM*	MG1655 *rho** Δ*aroM*	Present work
*rho**/Δ*visC*	MG1655 *rho** Δ*visC*	Present work
*rho**/Δ*apaH*	MG1655 *rho** Δ*apaH*	Present work
*rho**/Δ*ynbE*	MG1655 *rho** Δ*ynbE*	Present work
*rho**/Δ*yaaI*	MG1655 *rho** Δ*yaaI*	Present work
*rho**/Δ*ybaM*	MG1655 *rho** Δ*ybaM*	Present work
*rho**/*rpsL**	MG1655 with *rho** and *rpsL* nonsense mutation (*rpsL**)	Present work
