# Supplementary material for: Fitness Landscape Transformation through a Single Amino Acid Change in the Rho Terminator
Source: PLoS Genet. 2012 May 31;8(5):e1002744. doi: 10.1371/journal.pgen.1002744 (PMC3364947; doi:10.1371/journal.pgen.1002744)
Supplement: Table S9 — Primer pairs used over the course of the current study. (PDF) [file pgen.1002744.s018.pdf]

Table S9: Primer pairs used over the course of the current study.

Name	Forward primer	Reverse Primer	Usage
rho_kantag_U	AGGGAATTTCATGTTCGGGT<br>GCCCCGTCGCTAAAAAGTGT<br>AGGCTGGAGCTGCTTC	ACACTAAGTTAGCATGACTC<br>ACGCCGGGCGTCATATGAAT<br>ATCCTCCTTAG	Generation of kan cassette to tag *rho* (inserted upstream of *rho*)
rho_kanU_verif	ACCAAAGTGGGTGCACTGTC	GCCAGAAATCTGTTCACTTCG	Verifcation of *rho* upstream kan tag
rho_kantag_D	TATCTGTAATCTTAATGCCG<br>CGCTGGCGATGTTAGGAAAA<br>TGTGTAGGCTGGAGCTGCTTC	CCTGTGCAAAAATTAACCATT<br>TGATATGCAAATTGCATAACC<br>ATATGAATATCCTCCTTAG	Generation of kan cassette to tag *rho* (inserted downstream of *rho*)
rho_kanD_verif	TCGAAATGATGAAACGCTCA	CACGAAGACCTTTATTCAGAAGG	Verifcation of *rho* downstream kan tag
tufA_kantag	GCCTGACAAATGAAGCATTTT<br>AAAAACAGAAACATTCATAGT<br>GTAGGCTGGAGCTGCTTC	TATTCATATTTAAAATATCAA<br>TTCAATTTAACATTTTAAACA<br>TATGAATATCCTCCTTAG	Generation of kan cassette to tag *rpsL∗*
tufA_kan_verif	CCAGCCCCATACCAATCATA	GGGCGTTGTTGCTAAAGTTC	Verification of *rpsL∗* kan tag
rho_seq	GGATGAAATCGCTGACGAAT	TAACATGCCAGCAAATTCCA	Sequencing of *rho*
rho_long_seq	GGATGAAATCGCTGACGAAT	CCTGTGCAAAAATTAACCATT	Sequencing of *rho*
visC_seq	TATCAGCAGCGTCGACAGAG	TATCAGCAGCGTCGACAGAG	Sequencing of *visC*
envZ_seq	CCTGGCGAAACTGTTTATCG	TCCATCGACGTGCAGATTT	Sequencing of *envZ*
yadM_seq	AACGGTCAGTCCAATACTGTCT	GCATTAAATGGCTCGAACAAA	Sequencing of *yadM*
yagM_seq	TGCACTCCGGTGTCATAAAT	CTCGCCCAAAACAAATCAAT	Sequencing of *yagM*
ykgL_seq	CTCTAACTGGCACTCGAACG	AAGAACGCCATCCAGACTGT	Sequencing of *ykgL*
ppdD_seq	CAGTAGCTCATGCGAAGGTG	TTTCTTGTCTTTCGCTGCAA	Sequencing of *ppdD*
yadN_seq	GCCATACAGGTTACGAAGCA	GGCGACCTAATGAACAATGG	Sequencing of *yadN*
sthA_seq	CGATGGGGTTGTTTATCTGC	CAATAAAACGTCAGGGCAAAA	Sequencing of *sthA*
aroM_seq	GGGATCTGCGTCACATTTTT	TGGAAAAGCCGATTGATTTC	Sequencing of *aroM*
rpsL_seq	TTTTACGCTGACCAATGACG	TGGCGGGATCGTTGTATATT	Sequencing of *rpsL*
apaH_seq	GATGCGCAGAACTCTCACAG	GTACACCAGCGGTGCAATC	Sequencing of *apaH*
