# Supplementary material for: Fitness Landscape Transformation through a Single Amino Acid Change in the Rho Terminator
Source: PLoS Genet. 2012 May 31;8(5):e1002744. doi: 10.1371/journal.pgen.1002744 (PMC3364947; doi:10.1371/journal.pgen.1002744)
Supplement: Table S10 — Abbreviations for nutrient sources and antibiotics used throughout the study. (PDF) [file pgen.1002744.s019.pdf]

Table S10: Abbreviations for nutrient sources and antibiotics used throughout the study.

**Abbreviation**	**Compound**
CML	Chloramphenicol
STP	Streptomycin
NOV	Novobiocin
TMP	Trimethoprim
$CoCl_2$	Cobalt (II) chloride
FOS	Fosmidomycin
RIF	Rifampicin
PHL	Phleomycin
BAC	Bacitracin
TET	Tetracycline
KAN	Kanamycin
AKG	α-keto glutarate
NADM	N-acetyl D-mannosamine
