# Supplementary material for: Fitness Landscape Transformation through a Single Amino Acid Change in the Rho Terminator
Source: PLoS Genet. 2012 May 31;8(5):e1002744. doi: 10.1371/journal.pgen.1002744 (PMC3364947; doi:10.1371/journal.pgen.1002744)
Supplement: Table S11 — Number of technical replicates used to calculate growth rates and other statistics for each strain/growth condition combination. One well on a 96 well plate is considered one replicate. Technical replicates for each entry were in all cases spread across at least two different plate reader runs on different days. The tabulated counts do not include pruned replicates; see Text S1 for details. †: replicates used for spline-based fitting. ‡: replicates used for effective growth rate. (PDF) [file pgen.1002744.s020.pdf]

Table S11: Number of technical replicates used to calculate growth rates and other statistics for each strain/growth condition combination. One well on a 96 well plate is considered one replicate. Technical replicates for each entry were in all cases spread across at least two different plate reader runs on different days. The tabulated counts do not include pruned replicates; see Supplementary Methods for details. †: replicates used for spline-based fitting. ‡: replicates used for effective growth rate.

**Strain**	**Media**	**Condition**	n
WT	M9t/acetate		33
*rho**	M9t/acetate		24
WT	M9t/AKG		53
*rho**	M9t/AKG		61
WT	M9t/AKG		38†
*rho**	M9t/AKG		53†
WT/Δ*sthA*	M9t/AKG		28
*rho**/Δ*sthA*	M9t/AKG		23
WT/Δ*aroM*	M9t/AKG		8†
*rho**/Δ*aroM*	M9t/AKG		21†
WT/Δ*ybaM*	M9t/AKG		23
*rho**/Δ*ybaM*	M9t/AKG		29
WT/Δ*yaaI*	M9t/AKG		24
*rho**/Δ*yaaI*	M9t/AKG		28
WT	M9t/arabinose		24
*rho**	M9t/arabinose		12
WT	M9t/glucose		276
*rho**	M9t/glucose		241
WT	M9t/glucose		266†
*rho**	M9t/glucose		239†
WT/Δ*visC*	M9t/glucose		12
*rho**/Δ*visC*	M9t/glucose		12
WT/Δ*envZ*	M9t/glucose		31
*rho**/Δ*envZ*	M9t/glucose		34
WT/Δ*yadM*	M9t/glucose		28
*rho**/Δ*yadM*	M9t/glucose		28
WT/Δ*yagM*	M9t/glucose		40
*rho**/Δ*yagM*	M9t/glucose		34
WT/Δ*ykgL*	M9t/glucose		46
*rho**/Δ*ykgL*	M9t/glucose		42
WT/Δ*ppdD*	M9t/glucose		13
*rho**/Δ*ppdD*	M9t/glucose		13
WT/Δ*yadN*	M9t/glucose		14
*rho**/Δ*yadN*	M9t/glucose		13
WT/Δ*sthA*	M9t/glucose		25
*rho**/Δ*sthA*	M9t/glucose		27
WT/Δ*aroM*	M9t/glucose		27
*rho**/Δ*aroM*	M9t/glucose		27
WT/Δ*apaH*	M9t/glucose		28
*rho**/Δ*apaH*	M9t/glucose		27
WT/Δ*iraP*	M9t/glucose		34
*rho**/Δ*iraP*	M9t/glucose		31
WT/Δ*yaaI*	M9t/glucose		19

Continued on Next Page…

Table S11 – Continued

**Strain**	**Media**	**Condition**	n
*rho**/Δ*yaaI*	M9t/glucose		21
WT/Δ*ybaM*	M9t/glucose		18
*rho**/Δ*ybaM*	M9t/glucose		8
WT	M9t/glucose	1.875 μg/mL CML	99
*rho**	M9t/glucose	1.875 μg/mL CML	78
WT/Δ*envZ*	M9t/glucose	1.875 μg/mL CML	40
*rho**/Δ*envZ*	M9t/glucose	1.875 μg/mL CML	40
WT/Δ*yadM*	M9t/glucose	1.875 μg/mL CML	28
*rho**/Δ*yadM*	M9t/glucose	1.875 μg/mL CML	38
WT/Δ*iraP*	M9t/glucose	1.875 μg/mL CML	42
*rho**/Δ*iraP*	M9t/glucose	1.875 μg/mL CML	36
WT/Δ*apaH*	M9t/glucose	1.875 μg/mL CML	39
*rho**/Δ*apaH*	M9t/glucose	1.875 μg/mL CML	34
WT	M9t/glucose	2.0 μg/mL STP	65
*rho**	M9t/glucose	2.0 μg/mL STP	107
WT	M9t/glucose	2.0 μg/mL STP	82†
*rho**	M9t/glucose	2.0 μg/mL STP	102†
WT/Δ*yagM*	M9t/glucose	2.0 μg/mL STP	15
*rho**/Δ*yagM*	M9t/glucose	2.0 μg/mL STP	57
WT/Δ*ykgL*	M9t/glucose	2.0 μg/mL STP	57
*rho**/Δ*ykgL*	M9t/glucose	2.0 μg/mL STP	81
WT	M9t/glycerol		27
*rho**	M9t/glycerol		17
WT	M9t/lactate		40
*rho**	M9t/lactate		32
WT	M9t/lactose		27
*rho**	M9t/lactose		19
WT	LB		85
*rho**	LB		78
WT/*rpsL**	LB		22
*rho**/*rpsL**	LB		22
WT	M9t/NADM		35‡
*rho**	M9t/NADM		41‡
WT	M9t/ribose		49
*rho**	M9t/ribose		41
WT	M9t/pyruvate		16
*rho**	M9t/pyruvate		11
WT	M9t/Tween20		14
*rho**	M9t/Tween20		8
WT	M9t/xylose		20
*rho**	M9t/xylose		10
WT	M9t/glucose	2.0 μg/mL KAN	21‡
*rho**	M9t/glucose	2.0 μg/mL KAN	20‡
WT	M9t/glucose	0.5 μg/mL TET	21‡
*rho**	M9t/glucose	0.5 μg/mL TET	20‡
WT	M9t/glucose	100 μg/mL BAC	12‡
*rho**	M9t/glucose	100 μg/mL BAC	14‡
WT	M9t/glucose	0.5 μg/mL PHL	16
*rho**	M9t/glucose	0.5 μg/mL PHL	21
WT	M9t/glucose	1.5 μg/mL RIF	31
*rho**	M9t/glucose	1.5 μg/mL RIF	30

Continued on Next Page…

Table S11 – Continued

**Strain**	**Media**	**Condition**	n
WT	M9t/glucose	2.5 μg/mL FOS	$21^{\ddagger}$
*rho**	M9t/glucose	2.5 μg/mL FOS	$14^{\ddagger}$
WT	M9t/glucose	0.5 μg/mL TMP	14
*rho**	M9t/glucose	0.5 μg/mL TMP	14
WT	M9t/glucose	2.5 μM $CoCl_2$	$14^{\ddagger}$
*rho**	M9t/glucose	2.5 μM $CoCl_2$	$14^{\ddagger}$
