# Supplementary material for: Fitness Landscape Transformation through a Single Amino Acid Change in the Rho Terminator
Source: PLoS Genet. 2012 May 31;8(5):e1002744. doi: 10.1371/journal.pgen.1002744 (PMC3364947; doi:10.1371/journal.pgen.1002744)
Supplement: Text S1 — Detailed methods, extended discussion, and supplementary results. (PDF) [file pgen.1002744.s021.pdf]

Supporting Text for
Fitness landscape transformation through a single amino acid change in the Rho terminator

Peter L. Freddolino, Hani Goodarzi, and Saeed Tavazoie

Contents

1 Supplementary Methods **2**
1.1 Bacterial strains 2
1.2 Condition screening 3
1.3 Growth Curves 3
1.4 Transcriptome quantitation 5
1.5 Mutagenized library selection and transposon footprinting 6
1.6 Analysis of microarray data 7
1.7 iPAGE analysis 7
1.8 High throughput sequencing 8
1.9 Competition experiments 8
1.10 Statistical analysis 9

2 Alternative fitness metrics **11**

3 Validation of transposon library selection results **13**

4 Epistasis analysis **16**

5 Interaction of *rho and *rpsL**** **18**

References for Supporting Text **20**

1 Supplementary Methods

Media components and mineral oil were obtained from Fisher Scientific, and antibiotics and other added compounds from Sigma-Aldrich. All constructed strains had both their *rho* locus and any other targeted modifications confirmed by sequencing of gene-specific PCR products (Genewiz; South Plainfield, NJ) or electrophoretic analysis of PCR product sizes. PCR primers were obtained from Integrated DNA Technologies (Coralville, IA) and are listed in Table S9. For cloning and other routine procedures cells were grown in LB (10 g/L tryptone, 5 g/L yeast extract, 5 g/L NaCl) or on LB agar (LB plus 15 g/L agar). For physiological experiments cells were grown in M9 minimal media (6 g/L Na_2HPO_4, 3 g/L KH_2PO_4, 1 g/L NH_4Cl, 0.5 g/L NaCl, 1 mM $MgSO_4$) [1] supplemented with 3 mg/L $CaCl_2$, 0.5 g/L thiamine, and the micronutrient supplement described by Neidhardt *et al.* [2]; this base media composition is referred to as M9t. A final concentration of 0.2% glucose was provided as a carbon source unless otherwise noted. Throughout the paper we use the notation M9t/(*carbon source*) to refer to M9t with a given carbon source. For chiral carbon sources, we used the dominant biological form without further comment (D-glucose, L-arabinose, D-xylose, D-ribose, α-lactose). For carbon sources expected to require substantial amounts of iron for optimal growth (α-keto glutarate, xylose, N-acetyl D-mannosamine, succinate, pyruvate, Tween-20, acetate, lactate), ferric citrate was added to a final concentration of 400 nM.

1.1 Bacterial strains

All strains used in this study were derived from *E. coli* MG1655 [3], and are listed in Table S8. All single-gene knockouts were initially obtained from the Keio collection [4], transferred to our WT and *rho** backgrounds by P1vir transduction [1] and selection on LB+kanamycin plates (50 μg/mL), and then cured of their kanamycin resistance cassette using the helper plasmid pCP20 [5]. Transformed strains were incubated 24 hours at 42° C and then replica plated on LB, LB+kanamycin, and LB+ampicillin (100 μg/mL) to ensure loss of both the kanamycin cassette and pCP20. Knockouts were then confirmed by sequencing the target gene and the rho locus.

The *rho** strain was obtained by tagging the *rho* locus of a previously evolved ethanol tolerant strain [6] with a kanamycin resistance cassette (located 100 bp downstream of *rho*), followed by P1vir phage transduction into a clean WT background and pCP20-mediated excision of the resistance cassette (the procedure for cassette construction, incorporation, and excision follows that described by Datsenko and Wanner [7]; the rho_kantag_D primers from Table S9 were used for tagging *rho*). Separate testing of a rho^{WT} strain containing an equivalent scar from resistance cassette excision showed no significant difference in growth rate from the parental strain under any of the conditions in this study (data not shown), confirming the neutrality of the marker. A subset of the *rho** phenotypes were also tested in a second strain with the resistance cassette inserted at a different location (located 250 bp upstream of *rho*; generated using rho_kantag_U); no detectable differences were found between strains constructed using these methods.

1.2 Condition screening

In order to obtain a landscape of possible fitness differences between WT and *rho** cells, we took growth curves for both strains on Biolog Phenotype Microarrays 1, 3B, and 4A ($\sim$300 conditions in all). To start the plates, cells were grown overnight in M9t/glucose, pelleted, resuspended in six volumes of phosphate buffered saline (or 150 mM NaCl, in the case of plate 4A), mixed, and then diluted a further six-fold into Biolog IF0 (without addition of the redox-sensitive dye normally used in such plates). Each well was inoculated with 100 μL of this mixture, and growth curves were then measured as described above, except that the plate lid was used instead of mineral oil. Possible differences between WT and *rho** were identified by visual inspection of the growth curves. We identified 18 conditions of interest on the basis of Biolog plate screening, of which seven were chosen for follow-up. Five of the conditions chosen on the basis of this screening showed substantial WT/*rho** differences during follow-up experiments, and are included in Table 1.

We also screened approximately 35 additional conditions, including a variety of other common carbon sources and laboratory antibiotics, conditions which had yielded mutations in rho during directed evolution experiments [8–10], conditions chosen on the basis of expression microarray results, and a few conditions chosen based on our experience with the *rho*/rpsL** double mutant. Those conditions showing substantial differences in behavior from our baseline (M9t/glucose) are shown in Fig. 1 and Table 1; the remainder generally were indistinguishable from the reference condition in terms of *rho**/WT growth rate ratio.

1.3 Growth Curves

Bacterial growth curves were measured in Costar 96-well clear polystyrene plates, using either a Biotek Synergy MX or Powerwave XS2 plate reader (Biotek; Winooski, VT). Plates were incubated with continuous shaking, and optical density (OD) reads at 600 nm taken every 10 minutes. For all growth curves the wells contained 150 μL media, and were covered with 100 μL mineral oil. All experiments were carried out at 37° C unless otherwise noted. Cells for growth curves were pre-grown overnight in M9t/glucose (or in LB, for some cases where growth curves were to be taken subsequently in LB), and then diluted 200-1000 fold into target media. In cases where the target media contained nonstandard nutrient sources, the overnight culture was pelleted and resuspended in 150 mM sodium chloride or phosphate buffered saline prior to inoculation. All growth curves performed using mineral oil also had 0.001% Tween-20 (Sigma) added to the media to prevent aggregation-related artifacts. At least two biological replicates were performed on different days for each measurement, with plate positions randomized or perturbed between days (on each day, each cell/media pairing was run in either 7 or 11 independent wells). The total number of replicate measurements for each strain/condition combination is given in Table S11. All data from a total of three runs (out of $\sim$100) were excluded because all strains showed growth rates 30-50% different from typical rates for the same antibiotics, suggesting a problem with media preparation on those days.

Growth rates were calculated using a multistep procedure: blank-corrected ODs were $\log_2$ transformed; all data points with a blanked OD less than 0.001 were removed; simple heuristics were used to remove all portions of the growth curve containing bubbles or other

optical artifacts, based on the assumptions that the growth rate could not exceed 4 doublings per hour and that the lowest OD would occur at the first non-bubble timepoint. A linear regression on the data for which the $\log_2$ (blanked OD600) was between -6 and -4 was used to calculate the growth rate; only replicates for which this fit yielded an $r^2 > 0.998$ were retained for further analysis. The OD range used here was chosen to be at least 3-4 doublings after inoculation (and usually even more). In a small number of cases where the initial inoculum was larger, the two-doubling range for fitting was moved without comment. The slope within the fitted region for each technical replicate was then taken to be the growth rate for that replicate.

The overall growth rates for strain/media combinations were then calculated by applying a linear mixed-effects model to the logarithms of the growth rates, with modeling done separately for each media type. Prior to modeling, all points which were more than 2.5 times the interquartile range away from the median for a given strain/media combination were dropped as outliers; in virtually all cases these represented optical artifacts due to bubbles that escaped the filtering described above. The model then fitted the logarithm of the growth rates (measured as described above) with fixed effects for the strain and plate reader being used, and random effects for the plate ID (*i.e.*, the specific plate reader run) and for the strain identity within each plate. Effects were omitted as appropriate if there were not enough categories to fit the model (for example, if all runs for a given media type were on the same plate reader, the reader effect was omitted). We fitted the model using the `lme4` package [11] of R with a restricted maximum likelihood fit. Uncertainties and confidence intervals were obtained from 10,000 simulated draws from the estimated posterior distribution of model parameters, using the `sim` function of the `arm` R package [12] when possible, and the `mcmcsim` function of `lme4` when the former approach failed due to singularity in the parameter variance-covariance matrix. When `mcmcsim` was used we applied a burn-in period of 5,000 steps prior to sampling.

In a few cases (noted in Table 1), the fitting described above proved impossible; in these cases the cells followed consistent but unusual patterns, in which a rapid initial burst of growth occurred for one or two doublings, followed by a lag, and then a slower period of sustained long-term growth. As an example, data from a typical growth curve in M9t/NADM are shown in Figure S9; the advantage held in this media by *rho** cells is visually apparent. In order to provide a quantitative metric as close in spirit as possible to the growth rates used for other conditions, for growth on these media types we calculated an effective growth rate as follows: we smoothed the $\log_2$-transformed optical density with a cubic B-spline [13] and then calculated the effective growth rate (*i.e.*, average number of doublings per hour) during growth between $\log_2$ ODs of -5.5 – -3.5 (M9t/NADM), -5.0– -3.5 (M9t/glu + FOS), or -6 – -4 (others). In all cases we selected optical density ranges corresponding to the early post-burst phase of growth. All values given for the affected media types in tables or figures referring to a "growth rate" in fact refer to this effective growth rate (error analysis is performed exactly as described above for our other growth rate data).

The significance of growth rate differences between rho^{WT} and *rho** cells was assessed using glucose minimal media as a reference; the ratio of the *rho** and WT growth rates for a given condition was compared to that for M9t/glucose, and lack of overlap of the 95% confidence intervals taken to indicate significance.

During the validation of selection results described in Section 3, we also encountered some

mutants with atypical growth curves which did not provide log phase growth throughout the targeted optical density range. In order to avoid having the (sometimes small) differences in growth rate between these strains hinge upon manual selection of fitting ranges, in these cases a smoothing B-spline [13] was fitted to the remaining points and used to identify the time point of maximal slope (*i.e.*, specific growth rate). A linear regression was then applied to a one hour window centered on that point of maximal growth rate, and then expanded both forward and backward in time as far as possible while maintaining an $r^2 > 0.998$ for the linear fit. Data points occurring prior to four doublings of the initial optical density, or for which the blank-subtracted OD600 was less than 0.022, were ineligible for inclusion in fitting. This approach is referred to as spline-guided fitting, and was applied as noted in Table S4, Table S11, and Table S2. Spline-guided fitting applied to growth curves which were fit well using our baseline method yielded equivalent relative growth rates between strains but generally with larger within-day variations. In order to maintain consistency, we applied spline-guided fitting for any given knockout in both the rho^{WT} and *rho** backgrounds (and in all media conditions) if it was required for either.

For the purposes of Section 2, lag times were calculated as the time point at which a line tangent to the point at which the maximum growth rate occurred on a plot of $\log_2$ OD vs. time, with slope equal to the maximum growth rate, reaches the initial OD of the culture. The time to saturation was taken to be the difference between the timepoint at which the highest measured OD occurred and the lag time of the corresponding replicate (ignoring cases where the culture had not saturated during the measured time window). For both of these quantities, due to their heavy dependence on the exact state of the initial inoculum and early handling of the cultures, we considered only data points where the cell types being compared were both present on the same plate. We note that because our primary object of interest was the steady state growth rates, the present data are not ideal for determining lag times due to minor day to day variations in the time required for plate preparation; nevertheless, cases showing strong differences in lag time between rho^{WT} and *rho** cells could still be detected. Model fitting and error analysis for both of these quantities was performed as described above for growth rates, but with the model fit directly to the lag time and saturation time rather than the logarithm of those quantities (and using the same outlier-filtered set of technical replicates as used for the growth rate calculations).

1.4 Transcriptome quantitation

Relative transcript abundances in *rho** and WT cells were determined using a two-color hybridization protocol for Agilent arrays. Cells were grown overnight in M9t/glucose media. The cells were then diluted down to an OD600 of 0.02 in fresh M9t/glucose and were grown to an OD600 of 0.5. Total RNA was then extracted using a total RNA purification kit (Norgen Biotek, Cat 17200). A poly-A tail was added to the RNA samples using *E. coli* Poly(A) polymerase (New England Biolabs, M0276) for 15 minutes. Using an Agilent low input quick amp labeling kit, the *rho** and WT samples were then labeled with Cy3 and Cy5, respectively, and hybridized to Agilent custom arrays tiling the whole genome at 50 bp intervals, alternating between strands (Design ID 024568).

1.5 Mutagenized library selection and transposon footprinting

In order to obtain information on the fitness landscape under conditions showing differences in fitness between WT and *rho** cells, we performed selections on a transposon-mutagenized library under four different conditions in both genetic backgrounds (a schematic of the selection procedure is shown in Fig. 2A of the main text, and the selection conditions used are summarized in Fig. 2B). These selection experiments were motivated by the possibility that any given mutation may show differing effects in WT and *rho** backgrounds under some conditions, providing insight into the specific mechanisms through which *rho** alters the cell's regulatory and physiological state. A transposon insertion inside a gene generally disrupts the coding sequence and results in a null mutation; thus, upon selection, the fitness contribution of a given locus can be measured using the frequency of transposon insertion events that target its sequence (relative to a suitable reference condition).

Transposon-mutagenized cells were prepared for both the rho^{WT} and *rho** backgrounds following the protocol described by Girgis et al. [14]; the libraries contained approximately $1.4 \cdot 10^6$ and $8.4 \cdot 10^5$ unique transformants for WT and *rho**, respectively. The appropriate libraries were pelleted, resuspended in phosphate buffered saline, diluted 500-fold into M9t/glucose (reference media) or appropriate target media and harvested after 16 hours of growth at 37° C with shaking at 250 rpm. All selections were performed and hybridized in duplicate. The total culture volume used was 25 mL for each replicate. The transposon footprinting protocol used to analyze selections on mutagenized libraries closely follows that of Girgis *et al.* [15]. In short, we extracted genomic DNA from the selected pools (DNeasy Blood & Tissue Kit, Qiagen). Each sample was then split in half, and each half digested with either HinP1 or MspI (New England Biolabs) for 3 hours at 37° C. After heat inactivation of the enzymes, the digestion products were combined and a Y-shaped linker was ligated to the DNA fragments. The samples were then purified using QIAquick PCR purification columns and used as the template in a PCR containing a primer specific to the transposon and a primer specific to the Y-shaped linker. The T7 promoter on the transposon was used for *in vitro* transcription and labeling (Megascript®; Ambion). Libraries grown in M9t/glucose were used as controls and were labeled with Cy5; whereas the target selections were labeled with Cy3. The labeled samples were then hybridized to Agilent custom arrays tiling the whole genome (Design ID 024568).

We then sought to identify genes and pathways which showed significant enrichments or depletions of transposon insertions in the WT and/or *rho** backgrounds. We flagged as significant any location which shows both a significant Z-score (see below) and for which the smoothed log ratio of transposon insertion signals in the selected vs. unselected conditions indicated a greater than 1.5-fold change in abundance; these criteria establish technical and biological significance, respectively. Each selection showed hundreds or thousands of distinct sites with significant insertion levels, occurring in dozens or hundreds of genes and intergenic regions. The number of genes and intergenic regions with significant probes in each selection are shown in Fig. 3 of the main text and in Table S3, along with the number of loci unique to each genetic background. In order to avoid inflation of the number of differences between WT and *rho** in this analysis due to thresholding artifacts, Table S3 also shows the number of unique loci calculated using a strict criterion, in which the set of loci containing significant probes under some condition/genotype pair is compared with those occurring

using a reduced selection signal threshold (1.2-fold change) in the other background. For the purposes of Fig. 3, we consider a given locus to show "similar" patterns of insertions if the site contained only beneficial insertions (*i.e.*, probes with increased abundance in the selected populations) in both backgrounds (rho^{WT} and rho^*), only deleterious insertions in both backgrounds, or insertions of both types in both backgrounds. For the iPAGE data (third panel of Fig. 2D), correlation between pathway-level patterns for WT and rho^* cells was assessed using the Spearman ρ for their iPAGE over/under-representation patterns in the 15 bins used to quantize the data; the patterns were labeled as similar only if they were significantly correlated ($p < 0.05$ based on resampling). In no cases among these categories did the WT and rho^* data show anticorrelation (i.e., Spearman $\rho < 0$).

1.6 Analysis of microarray data

Spot intensities were extracted using Agilent Feature Extraction v.9.5. Spots were filtered using the IsSaturated, IsFeatNonUnifOL, IsPosAndSignif, and IsFound flags; any spots for which either of the former two were true, or either of the latter true were false, were discarded. All data were converted to log ratios of the appropriate sample and reference channels for each hybridization prior to analysis. In addition, the model-based LogRatioError for each spot was retained. Log ratios and errors for replicate spots both within each array and between duplicate arrays for each experiment were combined using the error-weighted combination method described in Section 3.1 of Weng *et al.* [16], providing a single estimated log ratio and LogRatioError for each probe. In addition, probe-wise Z-scores were calculated as the ratio of those two quantities for each probe location.

Z-score based significance calling was performed using Eq. 21 of Weng et al. [16]: for a Bonferroni-corrected p-value p, each probe i was considered significant only if its Z-score z_i satisfied the inequality

$$\mathrm{erfc}^{-1}(p) \cdot \sqrt{2} > \|z_i\| \tag{S1}$$

alongside additional log-ratio based criteria (described in the corresponding sections of the main text or Text S1). We used a p-value of 0.01 for all Z-score based significance calling.

During analysis of the selection results, we found in the case of the AKG selections that a small periodicity was present in the microarray data, centered on *oriC*; most likely, this indicates that the cells in the AKG selections either had not reached stationary phase or arrested in a different state than the reference selection with regard to genome replication. In order to remove the effects of this difference, we fitted a smoothing spline to the raw log ratio data with four knots (located at *oriC* and quadrants relative to it), and then subtracted the value predicted based on this spline from the log ratio at each probe (the model based errors were left unchanged). The correction was applied to all hybridizations of selected libraries for consistency, but was negligible for all cases except the AKG selections.

1.7 iPAGE analysis

Pathways showing significant enrichment or depletion of extreme RNA and selection values were identified using iPAGE 1.1 [17]. We used Gene Ontology (GO) terms [18] as a proxy for

pathways (annotations from revision 1.27, 2/16/2010), including only terms which contained at least two genes in the MG1655 genome. Each probe was assigned all GO terms of genes containing its center. During analysis of RNA data, we considered only probes which were on the sense strand of the coding region of a gene; for selection data, we considered all probes, with intergenic probes assigned the GO terms of the closest gene. In the RNA case, prior to iPAGE analysis, the unsmoothed log ratios were quantized into seven clusters, with cutoffs corresponding to 3, 2, and 1.5-fold changes (in either direction) in expression between WT and *rho** cells. For the selections (where there is no particularly informative choice of cutoff values), the unsmoothed log ratios were instead quantized into fifteen equally-populated bins. A p-value cutoff of 0.0001 was applied in significance testing; default values were used for all other settings. The results of the analysis were not qualitatively affected by altering the significance threshold by a factor of 10 in either direction, nor by allowing the reporting of redundant GO terms (data not shown).

1.8 High throughput sequencing

The parental strain and evolved ethanol-tolerant strain described by Goodarzi et al. [6] were sequenced following the published Illumina Genomic DNA sequencing protocol (Rev. B). Reagents for sample preparation were obtained as a NEBNext kit from New England Biolabs (Ipswich, MA). Barcoded adapters (using 3-base identifying sequences from Cronn et al. [19]) were used to enable multiplexing; the strains were then sequenced as part of a four-way multiplexed 101 bp single-end sequencing run on an Illumina HiSeq 2000. Sequencing results were divided by barcode (allowing only perfect matches) and adapter-only portions of reads removed using utilities from fastx_toolkit (`hannonlab.cshl.edu/fastx_toolkit/`). Surviving portions of reads were then aligned to the *E. coli* MG1655 reference genome (GenBank Accession U00096.2) using `bwa` [20], with a quality score cutoff of 25. Variant calling was then performed using the `samtools` [21] `pileup` and `varFilter` commands. The called variants were subsequently pruned by removing all variants with an aggregate SNP quality less than 20, or for which the variant was called solely on the basis of reads in one direction.

1.9 Competition experiments

As described above, evaluation of a true doubling time in M9t/NADM proved impossible due to the constantly changing specific growth rate exhibited during growth in this media; instead, an arbitrary time window was chosen within which an effective growth rate was evaluated to compare WT and *rho** cells. In order to avoid relying on this *ad hoc* definition while testing the effects of secondary mutations on growth in this media (as described in Section 3), we also evaluated the fitness of different strains through competition experiments. Similar experiments were used as an independent validation of observed epistatic interactions, as shown in Section 2.

For each experiment, cells of the strain of interest and a *lacZ*- strain (derived either from our rho^{WT} or *rho** strain by replacing *lacZ* with an antibiotic resistance cassette and inducible GFP marker) were grown for 24 hours in the media of interest (48 hours for growth in M9t/NADM), and then mixed in a 1:1 ratio. A suitable dilution (done serially by ten-fold increments in phosphate buffered saline) of the mixture was plated on indicator plates

(MacConkey agar plus 1% lactose w/v), and 3 mL of pre-warmed, pre-aerated target media inoculated with a 200-fold dilution of the mixture (600-fold for competitions in LB). After 18 hours of growth at 37° C with shaking at 250 rpm (48 hours of growth in M9t/NADM) the cultures were again diluted in PBS to an appropriate density and plated. All samples were diluted and plated in triplicate; two or more biological replicates were used for each strain/media combination.

For each biological replicate, the plate counts for each timepoint were pooled, and then used to calculate the realized Malthusian parameter for the competing strains (following [22]):

$$m_{\text{media,strain}} \equiv \log 2(n_{\text{final}}/n_{\text{init}})/t \tag{S2}$$

for a competition of duration t and for initial and final cfu counts n_{init} and n_{final} for a given strain. For each biological replicate of each competition, we then calculated the relative fitness ω of the competing strains (A and B),

$$\omega_{A,B} \equiv m_{\text{media,A}}/m_{\text{media,B}}. \tag{S3}$$

Both the multiplicative epistasis (Eq. S7) and the growth rate ratio (Eq. S4) may be expressed as a simple arithmetic function of these values of ω. We report quantities determined averaging over all relevant biological replicates for each ω. Error analysis was performed using Bayesian inference due to the small number of replicates. We modeled the observed values of ω for each biological replicate as being drawn from a Student-t distribution, with a mean specific to the media and pair of strains under competition, variance shared across all competitions in a given media type, and four degrees of freedom. For each media type, the means μ were assigned independent prior distributions uniform on $\log(\mu)$ between -3 and 3 (substantially exceeding the range of observed ω values), and the precision (inverse variance) assigned an uninformative $\Gamma(0.001, 0.001)$ prior. Posterior distributions were obtained via Gibbs sampling using JAGS [23], using three independent chains, with a burn-in period of 2,000 iterations followed by 10,000 samples. Convergence was assessed using the potential scale reduction factor [24, 25], which was less than or equal to 1.01 in all cases. Central 95% credible intervals for functions of the ω parameters, as well as posterior probabilities of specific values, were then calculated using the draws from the posterior distribution. For calculations of the growth rate ratio, we combined draws from the posterior distribution described here for fitnesses in M9t/NADM media with the simulated posterior distribution of growth rates in M9t/glucose to obtain the values needed for Eq. S4.

1.10 Statistical analysis

Data analysis was performed using custom written analysis tools in python or R, with scientific and statistical functions from Scipy [26] and R [27].

Mixed-effects model fitting and statistical analysis for the determination of growth rates from plate reader growth curves are described above in Section 1.3.

Mixed effects models for the manual growth curve data described in Section 5 were fitted using the R package `lme4` [11]. Restricted maximum likelihood fits were used in all cases. Growth rates were obtained from fitted strain-dependent fixed effects, with random effects

due to the day of the assay. Prior to fitting, all replicates were offset in time to yield an OD of 0.01 at time 0 (based on independent linear fits to each of them). Confidence intervals were obtained based on 10,000 simulations of the posterior distribution of the fitted model, using the R package `arm` [12], as described for the growth rate data in Section 1.3.

The significance of overlaps between probes flagged as enriched or depleted based on microarray analysis and annotated genomic features (*e.g.*, MDS42 sites) was assessed by generating 10,000 permutations in which the genomic features of interest were randomly repositioned (but not allowed to overlap); the p-value was taken to be the fraction of shufflings for which the magnitude of the log fold-change in overlap with the significant probes from the expected value was equal to or greater than that in the original data (this amounts to a two-tailed test for enrichment/depletion of overlap).

The gene-level significance of Z-scores obtained from selections on transposon-mutagenized libraries was assessed using a Monte Carlo permutation test: For each gene to be considered, the gene's median Z-score was taken as a summary statistic. We then generated 100,000 samples containing the same number of elements as the number of probes in the gene, drawn with replacement from the set of all probe-level Z-scores from the experiment of interest; the p-value was taken to be the fraction of samples with a median greater in magnitude than the magnitude of the gene's median Z score. We applied a Bonferroni correction for the 16 tests used in Table S2, and flagged as significant all gene/background combinations with a corrected p value less than 0.05.

2 Alternative fitness metrics

Throughout the main text, we use absolute growth rates as a proxy for fitness, for reasons both of principle and practice. Steady state growth rates offer a direct and reproducible reflection of the full extent to which cells of a given genotype are able to adapt themselves to a given environmental condition. Unlike other quantities which might be relevant to cellular fitness, steady state growth rates do not depend heavily on the exact state of the inoculum (unlike lag times) and do not vary over time (unlike stationary phase yields, which will vary differently for different strains as the cells begin to die off). In addition, the difference in growth rates between strains becomes increasingly dominant in their overall fitness as the number of generations undergone by populations in competition under reasonably constant conditions increases; that is, the relative growth rates will be asymptotically approached by the relative fitnesses obtained from direct competition experiments (the dominant alternative method for measuring fitness) as the number of doublings occurring during the competitions increases and the time the culture is left in stationary phase decreases.

Nevertheless, it may be of interest to consider other potentially relevant fitness metrics differentiating *rho** and rho^{WT} cells to see if they may show synergy or antagonism with the fitness effects due to differences in growth rate. The lag times and time to maximum yield for *rho** and rho^{WT} cells in a variety of media conditions are compared in Table S1 (the methods used to obtain growth curve-related quantities are detailed in Section 1.3). In principle, shorter lag times and more rapid growth to maximum yield, and higher yields at stationary phase will all contribute positively to overall fitness (with their relative importance depending on the details of how much time cells spend at steady state growth vs. in stationary phase or undergoing transitions between the two). As noted in Section 1.3, the protocol that we used to gather growth curves is not ideal for the measurement of lag times, but suffices in the case of major differences. We find that in all cases save one where significant differences in lag time or time to stationary phase were observed, they were synergistic with the fitness effects expected due to growth rate alone (in addition, several cases which narrowly miss our cutoff for statistical significance nevertheless are suggestive of the same effect, *e.g.*, lag time in M9t/arabinose or time to maximum OD in M9t/lactate). The one exception, lag times in M9t/Tween20, carries a high uncertainty but would dilute the advantage held by rho^{WT} cells in this condition during short competitions. The concordance between relative fitnesses obtained solely from consideration of growth rates with the contributions of lag time and rapidity of maximum yield suggest that measurements of growth rates alone are sufficient to detect the bulk of substantial phenotypic changes between rho^{WT} and *rho** cells. We observed no significant differences in maximum yield at stationary phase between rho^{WT} and *rho** cells in LB, M9t/glucose, or M9t/AKG, although in LB the *rho** cells appear to die off somewhat more quickly (data not shown).

As a similar test for our observations of epistasis combining the effects of lag time, exponential growth, and stationary survival, we repeated the epistasis measurements for four of the cases from Table S4 using head to head competitions as described in Section 1.9 (*n.b.* the genes showing epistatic interactions with *rho** in NADM-containing media had already been evaluated using head to head competitions as well). A comparison of the results from growth rates alone with head to head competitions is shown in Table S5. *visC* and *rpsL** show strong epistatic fitness effects in the direction expected on the basis of growth curve

data, and *aroM* also shows an epistatic interaction with *rho** of the correct magnitude and direction. In the remaining case, $\Delta yagM$, the competitions show weak or no epistasis whereas growth curves show a very strong positive epistatic interaction between *rho** and $\Delta yagM$. The discrepancy appears to arise due to differences in the susceptibility of the $\Delta yagM$ cells to streptomycin in the plate reader vs. bulk culture, as in identical media $rho^{\mathrm{WT}}/\Delta yagM$ cells do not even begin growing in the 18 hours alloted for the competition during growth curves taken on a plate reader (see Figure S4); differences could occur, for example due to different amounts of aeration between the conditions. The results obtained for this competition are also inconsistent with the very strong enrichment for transposon insertions in *yagM* observed during library selections in the rho^{WT} background (see Table S2).

It is also important to note that, unlike the case for analyzing strains that arise in laboratory evolution experiments, there is no "correct" set of parameters (initial dilution and competition duration) to use to assess the relative fitness of strains here, and the observed relative fitnesses will vary somewhat depending on those parameters – we have chosen values similar to those frequently used in directed evolution experiments, which allow for contributions to fitness from all phases of growth.

3 Validation of transposon library selection results

Transposon insertions such as those present in our library may exert their effects either by rendering a gene product nonfunctional (through insertions in the middle of an ORF), altering the regulatory logic governing a gene (through insertions in nearby regulatory elements), or by triggering direct overexpression of a gene from the promoter on the transposon's kanamycin resistance cassette; the first mechanism is generally dominant, and is the simplest to test. Direct identification of the identity and effect of a given selected insertion is further complicated by the fact that the selections occur in a mixed culture of a broad variety of mutagenized strains, whereas follow-up experiments are typically conducted in single strain or two strain cultures.

Both to provide validation for the selection results presented in the main text, and give concrete examples of loci for which disruption leads to distinct and different phenotypic changes in the WT and *rho** backgrounds, for each selection we ranked genes based on the median Z-score for all probes that they contained. For each condition, we then considered two genes which were among those with the largest (in magnitude) Z-scores for at least one of the genetic backgrounds, but did not correspond to a core metabolic pathway. To maximize the likelihood that the insertions identified in the selections corresponded to a loss of function in the target gene, we restricted ourselves to genes for which the Z-scores were uniform in sign within the genetic background showing significant changes. We then tested eight genes predicted by the selection experiments to show significant fitness effects by constructing in-frame deletions of each in the WT and *rho** backgrounds and comparing their growth with the corresponding parental strain.

The set of knockouts constructed for validation of the selection results is shown in Table S2. In all cases we compared the growth of strains in which the target gene had been deleted with the corresponding baseline (WT or *rho**), both under the selective condition of interest and our common reference condition (M9t/glucose). The observed intensity ratios from microarray data represent the ratio of the fitness of cells containing a given insertion under selective and unselective conditions; the most appropriate metric for validation of the microarray data, then, must compare the ratio of growth rates in selective vs. reference media for the mutant with those of the parental strain. For a given selective condition c, genetic background B (either WT or *rho**) and secondary gene deletion Δ, we define the growth rate ratio $\Gamma^c_{B,\Delta}$ as a function of the specific growth rates γ^c of individual strains to be

$$\Gamma^c_{B,\Delta} \equiv \frac{\gamma^c_{B,\Delta}/\gamma^{ref}_{B,\Delta}}{\gamma^c_B/\gamma^{ref}_B}. \tag{S4}$$

Values of $\Gamma^c_{B,\Delta}$ significantly above or below one indicate, respectively, that a given deletion is either beneficial or deleterious to growth in condition c relative to the reference condition.

In the case of growth in M9t/NADM, the above definition could not be used due to the atypical growth curves exhibited by most strains (see Methods for details). Instead, we performed competitions between the strains of interest and a common reference strain, and used the fitnesses (ω_A for each strain A, calculated as described in Section 1.9) in place of γ^c in Eq. S4; growth rates are still used for the reference media.

Of the nine background/deletion combinations present in our test set with significant selection Z-scores (based on the permutation test described in Section 1), eight also showed a significant change in growth rate ratio ($\Gamma^c_{B,\Delta}$ significantly different from one, judged by a two-tailed 95% confidence interval), confirming the strong influence of the target gene on growth under the condition of interest. One of the seven cases without significant selection Z-scores (WT/Δ*ppdD*) also showed a significant growth rate ratio, although the reason for the discrepancy with the selection results in this case is unclear (it is possible that no appropriately positioned transposon insertion was present in the library to mimic this deletion in the WT background).

Working under the (likely incorrect) assumption that all transposon insertions yield loss of function phenotypes of the gene that they are inside of or near, a negative selection score (*i.e.*, an insertion overrepresented in the selective media) is expected to correspond to a case where deletion of the affected gene yields a positive $\Gamma^c_{B,\Delta}$; the opposite is true for positive Z-scores. Deletions of *ykgL*, *envZ*, *aroM*, and *ppdD*, followed this pattern; the other cases (*yagM*, *sthA*, and *yadN*) showed the opposite pattern, presumably because the dominant transposon insertion leading to their selection in fact caused a gain of function phenotype in the corresponding gene.

We consider two specific cases in more detail below.

The effects of $\Delta yagM$ in WT and *rho** cells

As an example of a gene that appears likely to have different phenotypic effects in the WT and *rho** backgrounds due to direct differences in Rho-dependent termination, we consider *yagM*, a protein of unknown function contained in the CP4-6 prophage. As a prophage element with no annotated [28] or predicted [29] Rho-independent terminators, it is likely that regulation of transcriptional boundaries in this prophage is performed largely by Rho. Both sense and antisense RNA overlapping *yagM* is strongly up-regulated in *rho** relative to wild type cells; probe-level RNA data for the region are shown in Figure S5. Given that this region was also identified as a bicyclomycin-sensitive region (*i.e.*, one showing increased RNA polymerase occupancy after BCM treatment) by Peters and coworkers [30], the increase in RNA abundance likely represents a direct effect of transcriptional readthrough from *yagN* and *yagJ*. As seen in Figure S5, transposon insertions in and near *yagM* are heavily enriched in WT cells during growth in streptomycin-containing media relative to corresponding reference conditions, whereas the changes in *rho** cells are much less substantial.

Deletion of *yagM* has no measurable effect in either WT or *rho** cells in M9t/glucose, but as seen in Table S2, moderately inhibits *rho** growth and severely inhibits WT growth in the presence of streptomycin. The fact that in both WT and *rho** cells a transposon insertion in *yagM* was enriched in the presence of streptomycin, but that deletion of *yagM* in both cases leads to a reduction of growth on streptomycin, suggests that a transposon insertion present in the libraries causes some effect in *yagM* other than loss of function (either a gain of *yagM* function or a perturbation of transcription of *yagL*).

The effects of $\Delta aroM$ in WT and *rho** cells

In contrast with the *yagM* case, several genes showing substantial differences in transposon insertion frequency between WT and *rho** are not differentially expressed. For example, during growth of our transposon-mutagenized library on α-keto glutarate as a carbon source, insertions in *aroM* were strongly depleted in *rho** cells, but not WT cells. As seen in Figure S6, there is no significant change in expression of *aroM* in WT vs. *rho** cells (although the adjacent gene *yaiE* is overexpressed). $\Delta aroM$ cells showed a growth rate ratio of 0.982 in the WT background and 0.907 in the *rho** background, and thus the deletion shows the phenotype in both backgrounds that one expects on the basis of selection results. The mechanism through which *rho** interacts with $\Delta aroM$ is unclear; while it is tempting to assign the difference in behavior to effects on expression of the downstream *yaiE* gene, that explanation seems unlikely given that transposon insertions in *aroM* and wholesale deletion of *aroM* had the same effect on fitness.

4 Epistasis analysis

Given the breadth of Rho's regulatory scope, it appeared unlikely that *rpsL* would be the only gene showing epistatic interactions with the *rho** allele. We tested for epistatic interactions between *rho** and all eight gene deletions used in Section 3 (all but one of which showed selection scores suggesting an epistatic interaction), the *rpsL** allele described in Section 5, one additional gene deletion chosen based on its effects in the evolved HGDE3 strain (Δ *visC*), and four additional loci chosen by searching for the largest difference in Z-scores between WT and *rho** cells during the CML (Δ *apaH* and Δ *iraP*) and AKG (Δ *ybaM* and Δ *yaaI*) selections.

Conclusions regarding the presence or absence of epistasis for a given pair of genes are heavily dependent on the test statistic and null model used to evaluate potentially epistatic pairs [31, 32]. For all cases except that of growth on NADM, we use specific growth rates from plate reader experiments (calculated using the procedure in Section 1) as the fitness of each strain (for NADM-containing media, we instead used the procedure described in Section 1.9). We assume a multiplicative model for epistasis, as it is appropriate for use with growth rate data with small to moderate fitness differences between mutants and appears to be more robust than other definitions [31]. In the absence of epistatic interactions between genes A and B , the fitness ω_{AB} of an A/B double mutant is expected to follow

$$\omega_{AB} = \omega_A \omega_B \tag{S5}$$

where ω_i is the ratio of the growth rate (and thus fitness) of strain i to a common reference strain. The absolute epistasis ϵ_{AB} is then given by

$$\epsilon_{AB} = \omega_{AB} - \omega_A \omega_B \tag{S6}$$

In the present work, because growth rates (denoted γ_i for a given strain i) were the directly measured quantities, we obtain ϵ_{AB} from

$$\epsilon_{AB} = \frac{\gamma_{AB}}{\gamma_{WT}} - \frac{\gamma_A \gamma_B}{\gamma_{WT}^2}. \tag{S7}$$

In principle, reconsideration of this model for epistasis might be required for cases such as growth in the presence of antibiotics, where the definition above does not allow separation of changes in growth in a given growth medium from changes in resistance to a given antibiotic. In the cases considered here, however, no significant epistasis occurs in M9t/glucose (except, of course, for the Δ*visC* case).

As expected based on the genome-wide fitness landscape data described in the main text, we encountered several examples of epistasis during the course of our follow-up experiments, shown in Table S4 and in Fig. 4 of the main text. Each example showing significant epistasis illustrates a case where *rho** either masks or potentiates a fitness-affecting mutation. Note well that it is impossible to provide quantitative predictions for the value of $\epsilon_{A,rho*}$ for a given deletion A solely on the basis of the transposon-mutagenized library selection data, both due to the fact that the strains in the library do not match the in-frame deletions used in follow-up experiments, and because additional data would be necessary in the form of initial insertion abundances or quantitative cell titres before and after the selections.

Nevertheless, the selection Z-scores in the WT and *rho** backgrounds were either of different signs or widely separated by all but one (*envZ*) of the twelve loci that we considered on the basis of transposon library selections, qualitatively suggesting the presence of an epistatic interaction and motivating our use of this set of knockout strains to search for epistasis. Indeed, significant epistasis was observed in seven cases out of these 11 candidates.

5 Interaction of *rho** and *rpsL**

Upon construction of a clean *rho** strain through transduction of the HGDE3 *rho** allele, we found that *rho** by itself did not confer the level of ethanol tolerance observed in the evolved population. In order to identify other substantial contributors, we used the ADAM protocol [6] to perform global linkage analysis and find loci responsible for differences in ethanol tolerance between HGDE3 and *rho** (using LB + 5.5% ethanol as a selective condition and growth in LB as a reference condition). The resulting ADAM profile is shown in Figure S7A, and clearly indicates that a single additional contributor is primarily responsible for the difference, somewhere in the vicinity of *kefB* (which was also implicated, albeit more weakly, in the original comparisons of the parental and evolved strains, as shown in Figure S7B). We sequenced both the parental and evolved strains to greater than 750x coverage on an Illumina HiSeq 2000, and identified SNPs and small indels in both sequences relative to the MG1655 reference genome (raw sequencing data for the evolved strain are available from the Short Read Archive (SRA) as accession SRA045739.1). The only identified difference between the parental and evolved strains within 500 kilobases of the ADAM peak was a G→T substitution at position 3,472,415, which is a nonsense mutation leading to truncation of the S12 ribosomal protein (*rpsL*) after 59 amino acids (out of 159).

In order to test the effects of the evolved *rpsL* allele (henceforth *rpsL**), we constructed $rho^{\mathrm{WT}}/rpsL^*$ and *rpsL**/*rho** strains using methods equivalent to the *rho** strain, and analyzed their growth in the presence and absence of ethanol. Growth curves in ethanol-containing media cannot be conducted with the plate reader setup used throughout this study because the ethanol evaporates from the wells, yielding conditions that vary over time. Instead, growth curves were obtained manually in screw-capped 250 mL flasks; flasks were filled with 25 mL LB with 5.5% (v/v) ethanol added, heated/aerated for 20 minutes, and then inoculated with a 2500 to 5000-fold dilution of overnight culture of appropriate cells grown in LB. Equilibration and growth were at 37° C with shaking at 250 rpm. The cells were grown 20 minutes and then an OD600 measurement taken on an aliquot and used as an initial OD. Cells were subsequently grown with OD readings on further aliquots taken intermittently, and assumed to be in exponential growth after three doublings of the 20-minute optical density. Subsequent data points were $\log_2$-transformed and used in a linear fit to determine the growth rate. Four biological replicates on separate days were used for each strain (media for all strains on a given day was taken from a common pool); typical results from a single experiment are shown in Figure S8. Neither $rho^{\mathrm{WT}}/rpsL^{\mathrm{WT}}$ nor $rho^{\mathrm{WT}}/rpsL^*$ cells showed sustained growth beyond the three-doubling threshold, and thus fitting was only performed for $rho^*/rpsL^{\mathrm{WT}}$ and *rho**/*rpsL** cells. Fitted growth rates for all experiments are shown in Table S6; in addition, data from all experiments were offset in time to align the growth curves, and a linear mixed-effects model fitted to the composite data to yield strain-specific growth rates (using separate fixed effects for each strain, and date-dependent random effects shared across strains). The resulting growth rates were 0.85 doublings/hour ($rho^*/rpsL^{\mathrm{WT}}$) and 1.01 doublings/hour (*rho**/*rpsL**) and differed significantly ($p < 0.0001$ based on 10,000 draws from the posterior distribution of model parameters). In longer-term experiments, growth of $rho^{\mathrm{WT}}/rpsL^*$ cells eventually resumed 8-12 hours after inoculation, reaching a maximum rate of roughly 0.36 doublings/hour (data not shown); over the same period, growth of $rho^*/rpsL^{\mathrm{WT}}$ and *rho**/*rpsL** cells continued for several additional dou-

blings to saturating ODs between 0.3 and 0.6.

In light of the well-established role of Rho in mediating polar effects, the most plausible explanation for the non-additive interaction between *rho** and *rpsL** arises from the fact that rpsL occurs at the head of a transcript also containing *rpsG*, *fusA*, and *tufA* [33]. Thus, the introduction of a premature stop codon in *rpsL* (as in the *rpsL** allele) would be expected to decrease transcription of the remaining genes in the operon due to polarity; *rho** would, in turn, be expected to decrease the rate of early termination and thus increase transcript levels of *rpsG*, *fusA*, and *tufA* when introduced in the *rpsL** background.

*rpsL** and *rho** clearly exhibit negative epistasis during growth in LB (see Fig. 5). It is more difficult to state formally whether the phenotype of the double mutant in LB+5.5% ethanol represents a synergistic (*i.e.*, positive epistatic) interaction between *rho** and *rpsL**, given that none of the commonly used models for epistasis can identify epistatic effects in the presence of strains with near-zero fitness, and that the vast majority of literature dealing with epistasis is focused on pairs where at least one gene has a negative effect on fitness [31, 32, 34]. We consider *rho** and *rpsL** to exhibit positive epistasis here in light of the large fitness difference between $rho^{*}/rpsL^{\mathrm{WT}}$ and *rho*/rpsL** cells in ethanol, the negative epistasis observed in the absence of ethanol, and the clear mechanism for epistatic interaction between the mutations via decreased polar effects of *rpsL** in the *rho** background.

References

[1] Ausubel FM, editor (2001) Current Protocols in Molecular Biology. John Wiley & Sons, Inc.

[2] Neidhardt FC, Bloch PL, Smith DF (1974) Culture medium for enterobacteria. J Bacteriol 119:736–747.

[3] Blattner FR, Plunkett G, Bloch CA, Perna NT, Burland V, et al. (1997) The complete genome sequence of *Escherichia coli* K-12. Science 277:1453–1462.

[4] Baba T, Ara T, Hasegawa M, Takai Y, Okumura Y, et al. (2006) Construction of *Escherichia coli* K-12 in-frame, single-gene knockout mutants: the Keio collection. Mol Syst Biol 2:2006.0008.

[5] Cherepanov PP, Wackernagel W (1995) Gene disruption in *Escherichia coli*: TcR and KmR cassettes with the option of Flp-catalyzed excision of the antibiotic-resistance determinant. Gene 158:9–14.

[6] Goodarzi H, Hottes AK, Tavazoie S (2009) Global discovery of adaptive mutations. Nat Methods 6:581–583.

[7] Datsenko KA, Wanner BL (2000) One-step inactivation of chromosomal genes in *Escherichia coli* K-12 using PCR products. Proc Natl Acad Sci USA 97:6640–6645.

[8] Conrad TM, Lewis NE, Palsson BO (2011) Microbial laboratory evolution in the era of genome-scale science. Mol Syst Biol 7:509.

[9] Conrad TM, Joyce AR, Applebee MK, Barrett CL, Xie B, et al. (2009) Whole-genome resequencing of *Escherichia coli* K-12 MG1655 undergoing short-term laboratory evolution in lactate minimal media reveals flexible selection of adaptive mutations. Genome Biol 10:R118.

[10] Kishimoto T, Iijima L, Tatsumi M, Ono N, Oyake A, et al. (2010) Transition from positive to neutral in mutation fixation along with continuing rising fitness in thermal adaptive evolution. PLoS Genet 6:e1001164.

[11] Bates D, Maechler M, Bolker B (2011) Linear mixed-effects models using S4 classes.

[12] Gelman A, Hill J (2006) Data Analysis Using Regression and Multilevel/Hierarchical Models. Cambridge University Press.

[13] Dierckx P (1975) An algorithm for smoothing, differentiation and integration of experimental data using spline functions. J Comp Appl Math 1:165–184.

[14] Girgis HS, Liu Y, Ryu WS, Tavazoie S (2007) A comprehensive genetic characterization of bacterial motility. PLoS Genet 3:1644–1660.

[15] Girgis HS, Hottes AK, Tavazoie S (2009) Genetic architecture of intrinsic antibiotic susceptibility. PLoS One 4:e5629.

[16] Weng L, Dai H, Zhan Y, He Y, Stepaniants SB, et al. (2006) Rosetta error model for gene expression analysis. Bioinformatics 22:1111–1121.

[17] Goodarzi H, Elemento O, Tavazoie S (2009) Revealing global regulatory perturbations across human cancers. Mol Cell 36:900–911.

[18] Ashburner M, Ball CA, Blake JA, Botstein D, Butler H, et al. (2000) Gene ontology: tool for the unification of biology. the Gene Ontology Consortium. Nat Genet 25:25–29.

[19] Cronn R, Liston A, Parks M, Gernandt DS, Shen R, et al. (2008) Multiplex sequencing of plant chloroplast genomes using Solexa sequencing-by-synthesis technology. Nucleic Acids Res 36:e122.

[20] Li H, Durbin R (2009) Fast and accurate short read alignment with burrows-wheeler transform. Bioinformatics 25:1754–1760.

[21] Li H, Handsaker B, Wysoker A, Fennell T, Ruan J, et al. (2009) The sequence alignment/map format and samtools. Bioinformatics 25:2078–2079.

[22] Lenski RE, Rose MR, Simpson SC, Tadler SC (1991) Long-term experimental evolution in *Escherichia coli*. I. Adaptation and divergence during 2,000 generations. Am Naturalist 138:1315–1341.

[23] Plummer M (2011) JAGS Version 3.1.0 user manual.

[24] Gelman A, Rubin DB (1992) Inference from iterative simulation using multiple sequences. Statistical Science 7:457–511.

[25] Brooks SP, Gelman A (1998) General methods for monitoring convergence of iterative simu- lations. Journal of Computational and Graphical Statistics 7:434–455.

[26] Jones E, Oliphant T, Peterson P, et al. (2001–). SciPy: Open source scientific tools for Python.

[27] R Development Core Team (2010) R: A Language and Environment for Statistical Computing. R Foundation for Statistical Computing, Vienna, Austria.

[28] Keseler IM, Bonavides-Martnez C, Collado-Vides J, Gama-Castro S, Gunsalus RP, et al. (2009) Ecocyc: a comprehensive view of *Escherichia coli* biology. Nucleic Acids Res 37:D464–D470.

[29] Lesnik EA, Sampath R, Levene HB, Henderson TJ, McNeil JA, et al. (2001) Prediction of rho-independent transcriptional terminators in *Escherichia coli*. Nucleic Acids Res 29:3583–3594.

[30] Peters JM, Mooney RA, Kuan PF, Rowland JL, Keles S, et al. (2009) Rho directs widespread termination of intragenic and stable RNA transcription. Proc Natl Acad Sci USA .

[31] Mani R, Onge RPS, Hartman JL, Giaever G, Roth FP (2008) Defining genetic interaction. Proc Natl Acad Sci USA 105:3461–3466.

[32] Gao H, Granka JM, Feldman MW (2010) On the classification of epistatic interactions. Genetics 184:827–837.

[33] Saito K, Mattheakis LC, Nomura M (1994) Post-transcriptional regulation of the *str* operon in *Escherichia coli.* Ribosomal protein S7 inhibits coupled translation of S7 but not its independent translation. J Mol Biol 235:111–124.

[34] Phillips PC (2008) Epistasis–the essential role of gene interactions in the structure and evolution of genetic systems. Nat Rev Genet 9:855–867.

[35] Dornenburg JE, Devita AM, Palumbo MJ, Wade JT (2010) Widespread antisense transcription in escherichia coli. MBio 1.
